# Supplementary material for: Behavioral decomposition reveals rich encoding structure employed across neocortex in rats
Source: Nat Commun. 2023 Jul 4;14:3947. doi: 10.1038/s41467-023-39520-3 (PMC10319800; doi:10.1038/s41467-023-39520-3)
Supplement: Supplementary file 1 — Supplementary Information [file 41467_2023_39520_MOESM1_ESM.pdf]

Supplementary Material

Behavioral decomposition reveals rich encoding structure employed across neocortex in rats

Bartul Mimica[1ɫ*], Tuçe Tombaz[2ɫ], Claudia Battistin[3,2§], Jingyi Guo Fuglstad[2§], Benjamin A. Dunn[3,2] and Jonathan R. Whitlock[2*]

1. Princeton Neuroscience Institute, Princeton University, Princeton, NJ 08544, USA
2. Kavli Institute for Systems Neuroscience, Norwegian University of Science and Technology, NO-7030 Trondheim, Norway
3. Department of Mathematical Sciences, Norwegian University of Science and Technology, NO-7491 Trondheim, Norway

ɫ authors contributed equally to this work

§ authors contributed equally to this work

*corresponding authors: bmimica@princeton.edu, jonathan.whitlock@ntnu.no

Supplementary Figs. 1-24

Supplementary Tables 1-2

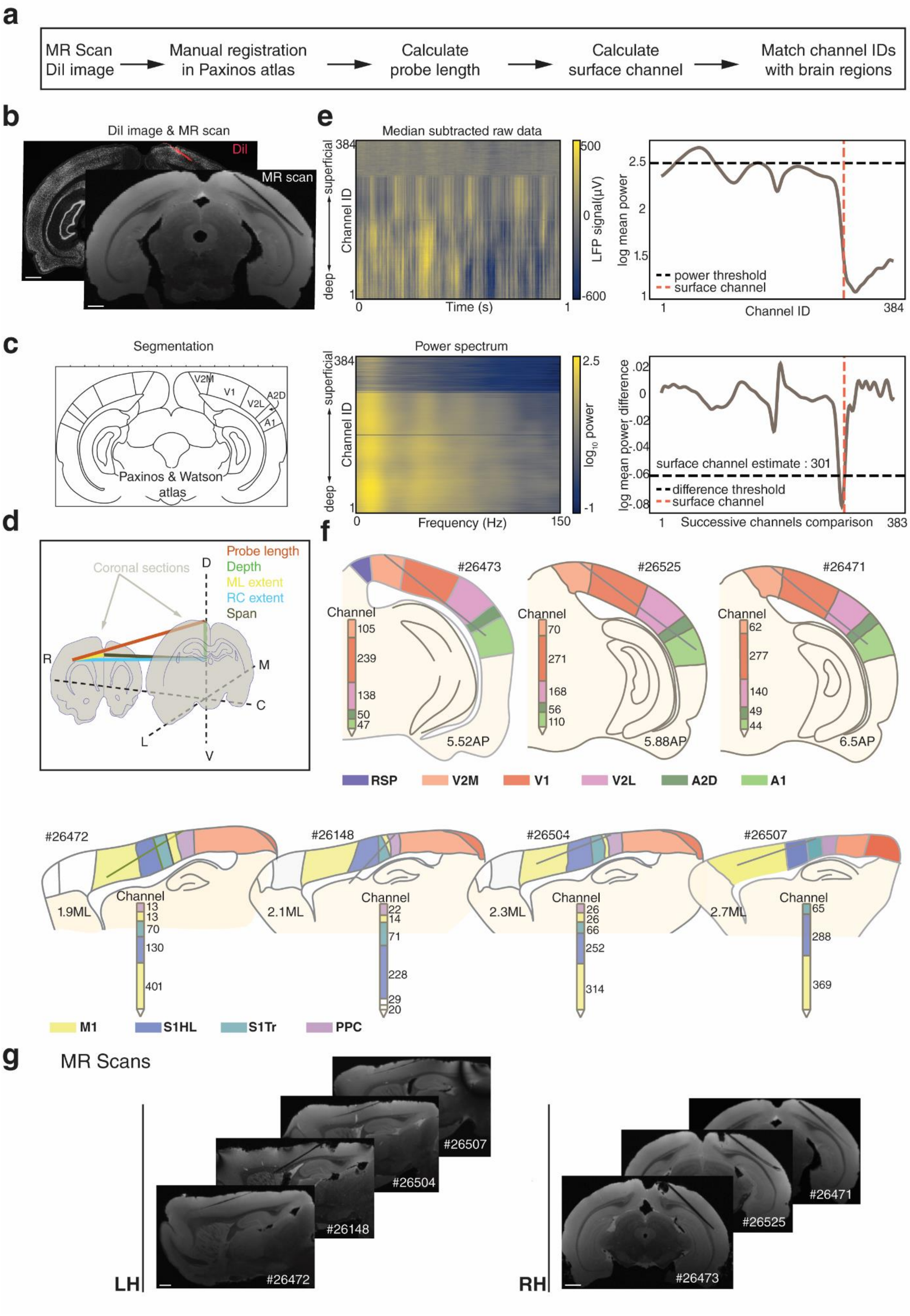
a
MR Scan
DiI image
Manual registration
in Paxinos atlas
Calculate
probe length
Calculate
surface channel
Match channel IDs
with brain regions
b
DiI image & MR scan
DiI
MR scan
c
Segmentation
V2M
V1
V2L
A2D
A1
Paxinos & Watson
atlas
d
Probe length
Depth
ML extent
RC extent
Span
Coronal sections
D
V
R
M
C
L
e
Median subtracted raw data
Channel ID
superficial
deep
Time (s)
LFP signal(μV)
log mean power
power threshold
surface channel
Channel ID
Power spectrum
Frequency (Hz)
log₁₀ power
log mean power difference
surface channel estimate : 301
difference threshold
surface channel
Successive channels comparison
f
#26473
#26525
#26471
Channel
5.52AP
5.88AP
6.5AP
RSP
V2M
V1
V2L
A2D
A1
#26472
#26148
#26504
#26507
1.9ML
2.1ML
2.3ML
2.7ML
M1
S1HL
S1Tr
PPC
g
MR Scans
LH
RH
#26472
#26148
#26504
#26507
#26473
#26525
#26471

Supplementary Fig. 1: Probe and channel localization in sensory and motor cortices. a, Pipeline for localizing Neuropixels 1.0 probes and recording sites in specific brain regions. **b,** Coronal section of a CM-DiI and NeuN co-stained section (back) and MR-scan of the same brain prior to sectioning (front) with a probe spanning visual and auditory cortices (similar sections were prepared for each of the 7 animals). Scale bar = 1mm. **c,** Atlas images (Paxinos & Watson, 2007) were used to determine regional boundaries for coronal sections containing CM-DiI-stained probe tracks. **d,** Schematic showing how probe placement (red) was registered in 3D space with respect to dorsoventral (DV; green), anteroposterior (AP; blue), and mediolateral (ML; yellow) axes. **e,** The surface channel was located using electrophysiological measures across all recording sites. (Upper left) The mean subtracted LFP signal and (lower left) power spectrum across recording channels change abruptly where the probe exits the brain (1 s of data shown). (Upper right) Power fluctuations across low band frequencies (<10 Hz) with an arbitrary threshold (black dashed line) and surface channel estimate (red) obtained from power differentials, below. (Lower right) Differences in power between successive channels with a $\log_{10}$ mean power difference threshold no higher than -.06 (black) indicate the surface channel as 301 (red). **f,** Anatomical reconstructions of probe placements based on probe length and channel count estimates. (Top row) Probe track reconstructions in the coronal sections from the brains of three rats with probes spanning primary and secondary visual and auditory areas in the right hemisphere; sections are arranged from anterior (left) to posterior (right). (Bottom row) Sagittal reconstructions of probe tracks in the left hemisphere of four rats; sections are arranged from medial (left) to lateral (right). Dark gray lines denote the probe location in each brain, and the number of channels in each region are shown on schematic probes inset with each reconstruction. **g,** MR scans showing probe locations in each of the seven animals. LH shows probe placement in primary somatosensory and motor cortices in the sagittal plane, whereas RH shows probe placement in visual and auditory regions in the coronal plane. Scale bars = 1mm.

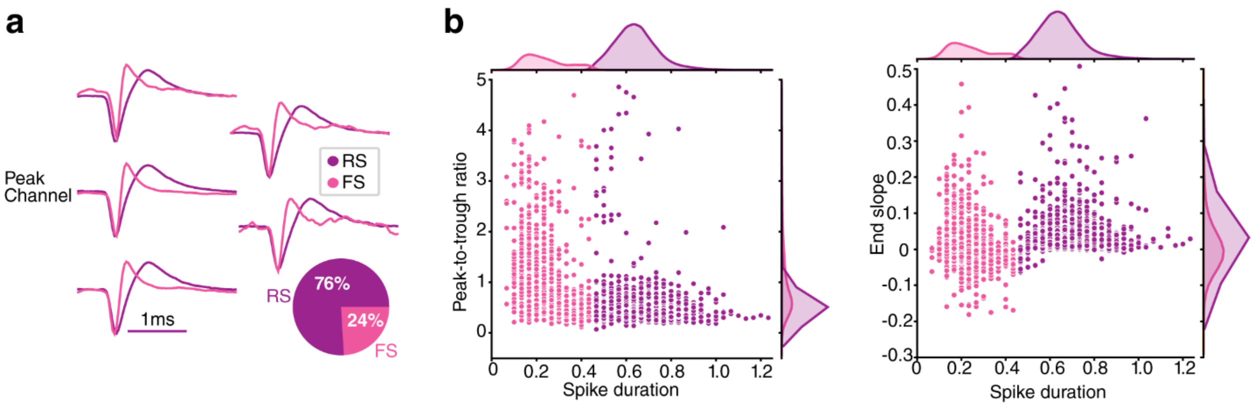

Supplementary Fig. 2: Classifying single units on the basis of spiking profiles. a, Mean waveforms of two example clusters on the same set of adjacent channels: fast spiking (FS) cluster in magenta, and regular spiking (RS) cluster in purple; (bottom right) overall breakdown of FS and RS clusters in the entire dataset. **b**, (Left) FS and RS peak-to-trough ratio and spike duration distributions. (Right) FS and RS end slope and spike duration distributions. Source data are provided as a Source Data file.

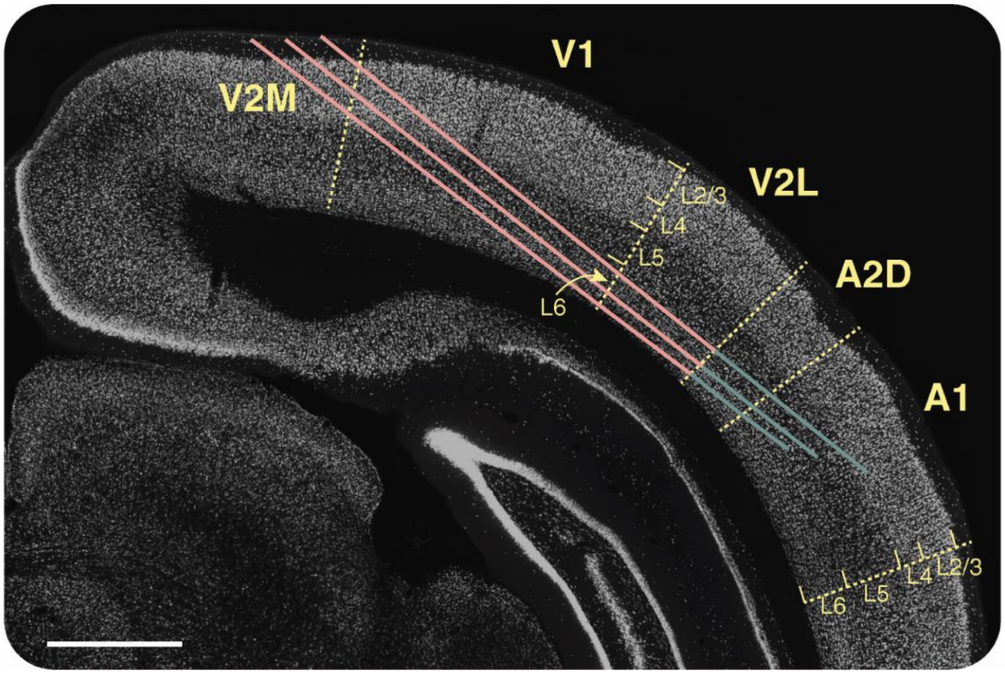

Supplementary Fig. 3: Laminar distribution of probes targeting visual and auditory cortices. Recording probes from the three right-hemisphere implanted animals are overlaid on a single reference section, displaying the putative cortical layers where single units were recorded in visual (pink) and auditory (cyan) areas. Probe placement was verified separately in each of the 3 animals. Scale bar = 1mm.

a

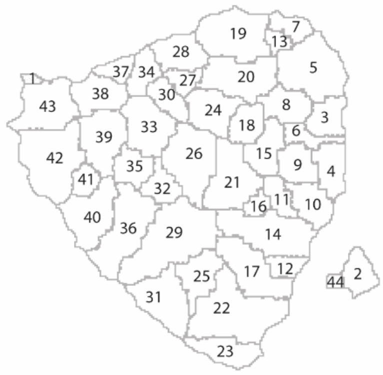

#	Actions	#	Actions
1	Still, sitting	23	Running, head up
2	Rearing, pre-rearing	24	Still, CW head roll, slight up
3	Turn left	25	Waddle, head centered
4	Slow walk, head left, CW head roll	26	Walk, CW head roll
5	Still, head down left, curl left	27	Still, CCW head roll
6	Slow walk, head left	28	Still, head level
7	Still, eating, back hunched	29	Walk, head-turn right
8	Still, head slight down left	30	Still, head up
9	Walk, head bobble	31	Head slight up, right
10	Walk, head up left	32	Walk, head & body turned right
11	Walk, head level, slight rightward movement	33	Still, head right, CW roll, movement
12	Waddle, head left	34	CCW roll, head moving slightly right
13	Still, head down left	35	Still, head right, CW roll
14	Walk, head scanning	36	Walk, slight head down right
15	Still, head level, left	37	Still, slight CCW head roll
16	Still, head scanning	38	CCW head roll, head down, moving right
17	Walk, slight head up, CW roll	39	Still, head right
18	Still, head slight left	40	Walk, head right, slight up
19	Still, back hunched	41	Still, head turn right
20	Still, CW head roll	42	Still, curled right, head down right
21	Walk, slight CW head roll, left head azimuth	43	Still, back hunched, head down
22	Running, head level, scanning	44	Rearing up

b

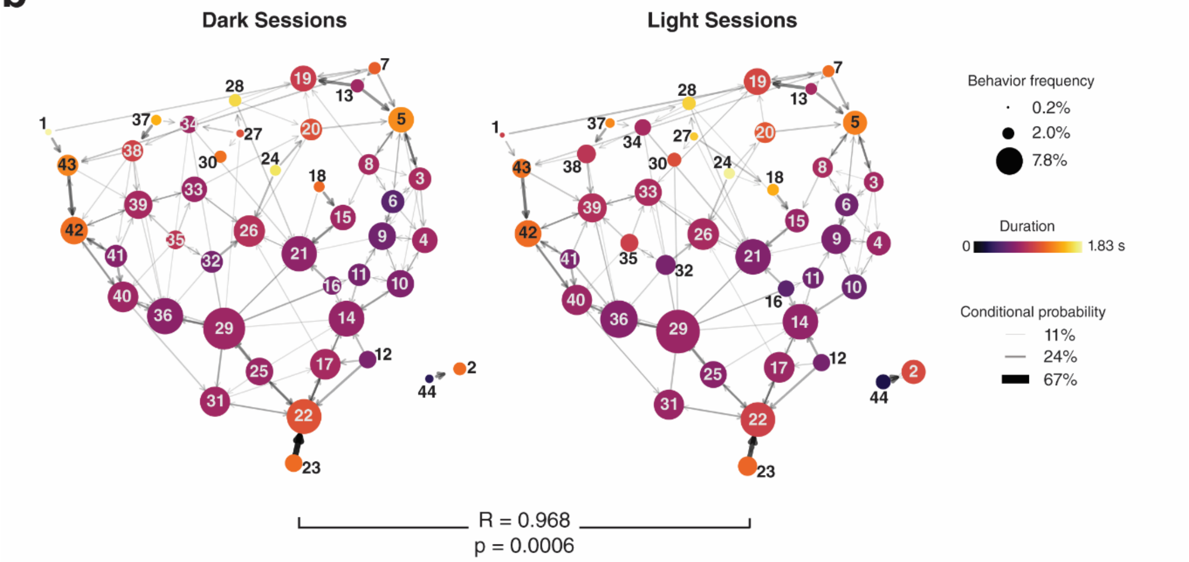

Supplementary Fig. 4: Description and statistics of extracted actions. a, Segmented t-SNE behavioral space with numerical labels and corresponding descriptions for each action. The actions embedded in t-SNE space followed a coarse inherent organization of rudimentary features. These included an increase in running speed progressing from the top to the bottom of the tSNE map, as well as a tendency for the back to be low or hunched at the upper-left of the map, and raised vertically at the lower right of the map (*i.e.* at the rearing actions, 2 and 44). Head pitch followed a similar coarse diagonal (head lowered in the upper left portions in t-SNE space and raised at lower and rightward regions). **b**, Ethograms displaying the frequency (shown via node size) and duration (shown via color) indicate mean action duration in light session was (0.47±0.33 s, mean ± SD across actions) and (0.49±0.36 s) in dark sessions. Time-conditional probabilities of subsequent actions are indicated by arrow transparency and thickness and were highly correlated across dark and light recording sessions (Pearson's correlation coefficient and p-value for 2-sided t-test shown beneath the matrices). Source data are provided as a Source Data file.

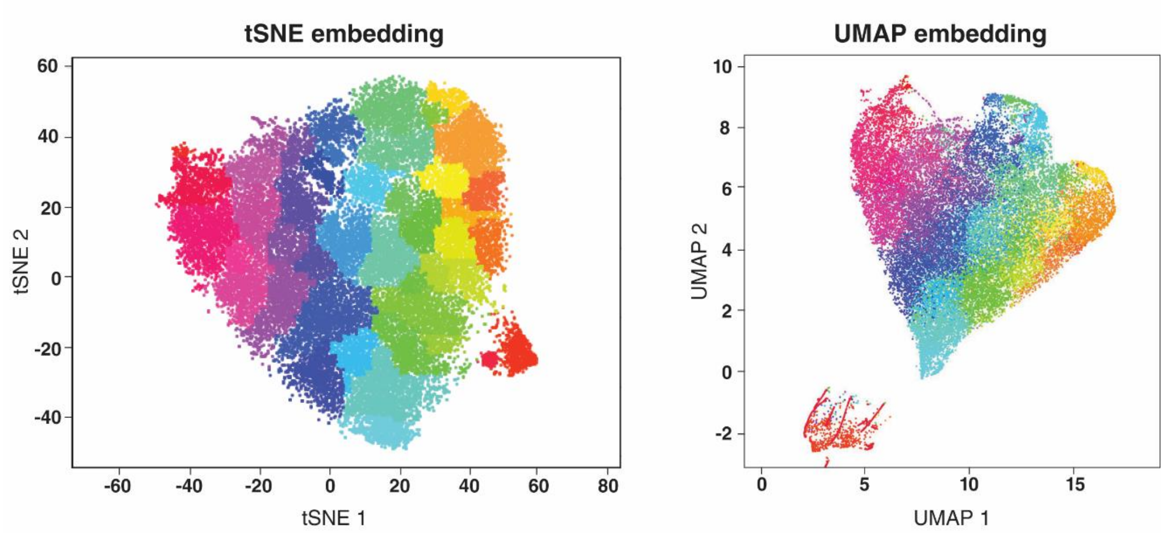

Supplementary Fig. 5: Comparison of low-dimensional embedding using t-SNE and UMAP. During peer review, t-SNE and UMAP embeddings of the tracking data were generated to determine if one of the methods allowed for better inter-subject identification of discrete actions. To compare methods, the watershed color-coded scatter plot of training points used for the t-SNE embedding (left) were embedded in 2D with UMAP (right). t-SNE and UMAP embeddings performed similarly in preserving both local structure (*i.e.* tSNE-classified points were clustered together in the UMAP space) as well as global structure (*i.e.* the gradient of color in the two embeddings was the same, with the exception of “rearing” and “rearing up”, in red). We tested whether the distributions were uncorrelated using the Jaccard’s similarity for the k-nearest neighbors. At both micro (k=2) and meso (k=100) scales, the p-value associated with the median of the Jaccard’s similarity was p = 0.001 under a 2-sided randomization test, indicating that UMAP and tSNE embeddings were very unlikely to be uncorrelated.

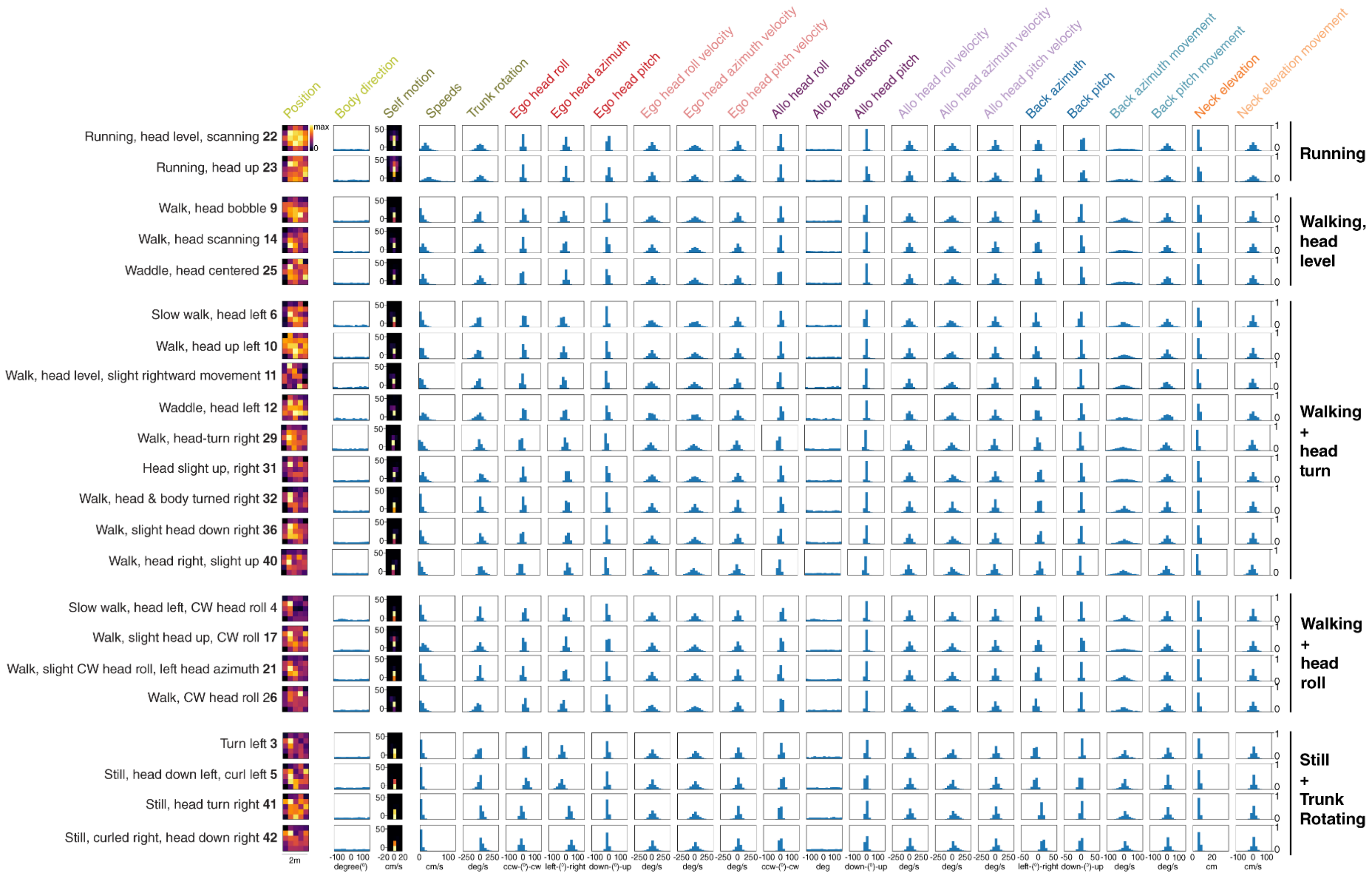

Supplementary Fig. 6a: Postural and kinematic composition of each of the 44 defined actions. Distributions of 23 spatial, postural and movement variables (Methods) for each of 22 actions, identified by the descriptor and action number on the left. Actions are grouped by similarity with regard to movement states (*e.g.* running, walking) and features of the head (*e.g.* turned) or trunk (*e.g.* hunched). 2D variables (position and self-motion; occupancy scaled from low (black) to high (yellow)) are expressed in 5x5 bins, and 1D variables in 15 bins. Source data are provided as a Source Data file.

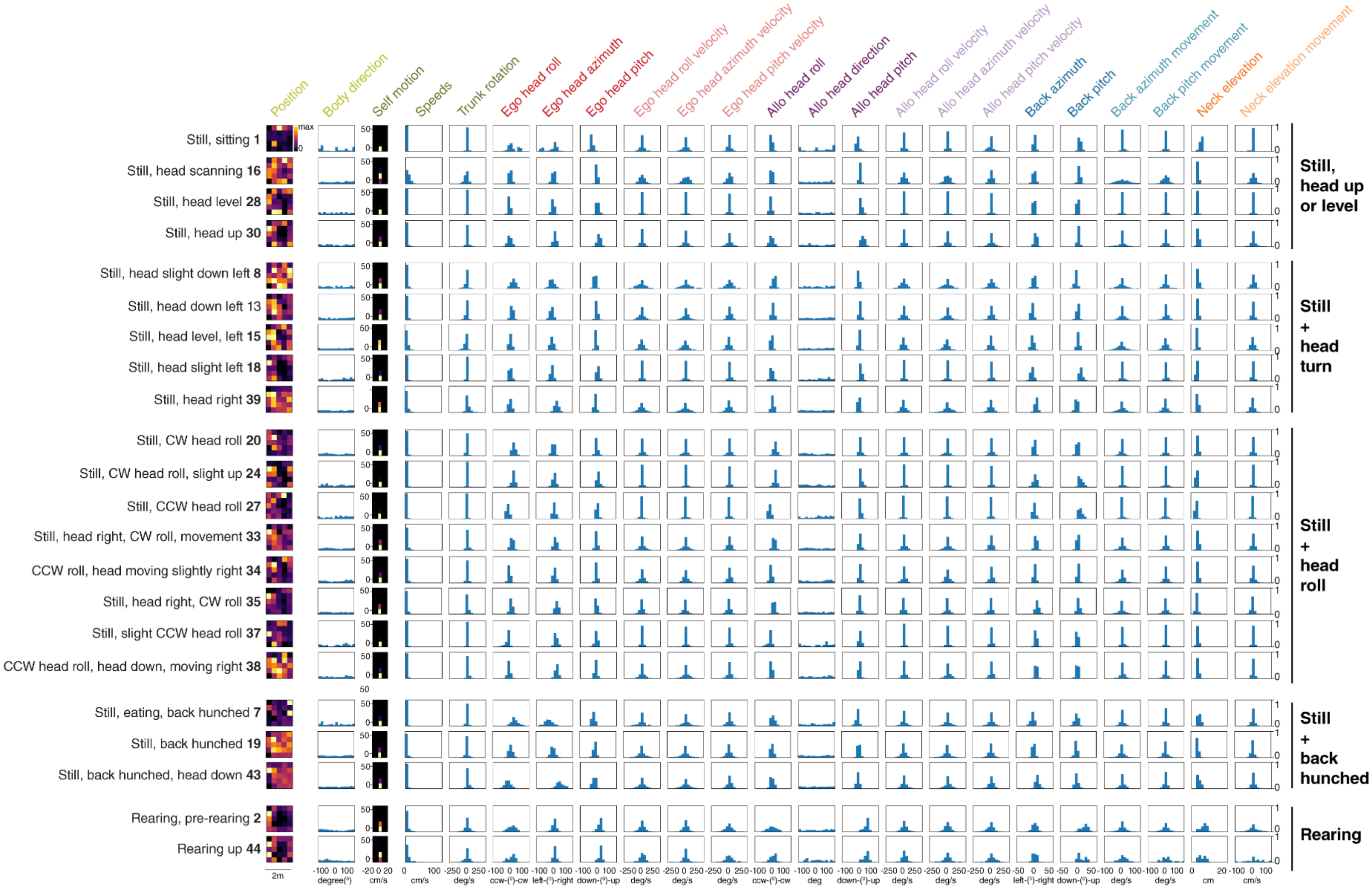

Supplementary Fig. 6b: Postural and kinematic composition of each of the 44 defined actions. Distributions of 23 spatial, postural and movement variables (Methods) for an additional 22 actions, identified by the descriptor and action number on the left (as in 6a). Actions are grouped and binned the same as in Supplementary Fig. 6a. Source data are provided as a Source Data file.

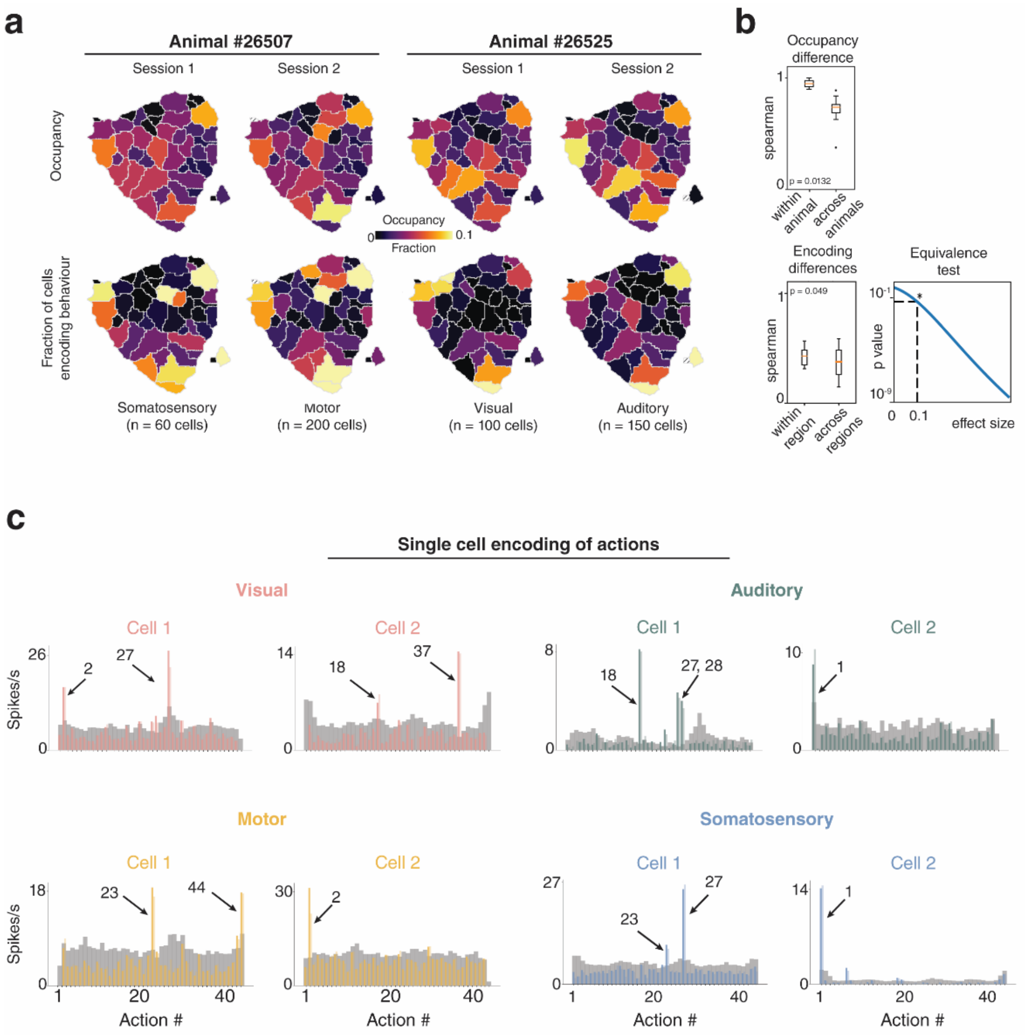

Supplementary Fig. 7: Action encoding across cortical areas. a, (top) t-SNE maps showing the relative occupancy of actions over two recording sessions from two example animals. (bottom) Fraction of cells encoding each action from the same example sessions (see Methods). **b**, (top) Spearman's ρ of the relative occupancy of actions between sessions for the same animal, 0.94 ± 0.06 (mean ± standard deviation (SD); n = 10; box plots extend from the 25th to 75th percentile of the sample distribution; orange lines indicate the median; whiskers span the data range; outliers (black dots) were excluded), and between sessions of different animals, 0.71 ± 0.13 (mean ± SD; n = 68; p = .0132, 1-sided Mann-Whitney U test for difference between medians in the two groups). (bottom left) Spearman's ρ of the fraction of cells encoding each action between recordings from the same brain region 0.40±0.14 (mean ± SD; n = 38; p = .004, permutation test), and between recordings from different brain regions 0.36±0.16 (mean ± SD; n = 98; p = .008, permutation test). (bottom right) p-value of two 1-sided t-test statistics for the equivalence between means of the Spearman's ρ in the two groups as a function of effect size (Cohen's d; *: p = .049 at Cohen's d effect size=0.1). **c**, Examples of actions encoded in visual (pink), auditory (cyan), motor (yellow) and somatosensory (blue) cortices. Cell 1 in visual cortex encoded "rearing, pre-rearing" (action #2) and "still, CCW head roll (action #27); Cell 2 encoded "still, head slight left" (#18) and "still, slight CCW head roll" (#37). Cell 1 in auditory cortex encoded "still, head slight left" (#18), "still, CCW head roll" (#27) and "still, head level" (#28); Cell 2 encoded "still, sitting" (#1). In motor cortex, Cell 1 encoded "running, head up" (#23) and "rearing up" (#44); Cell 2 encoded "rearing, pre-rearing" (#2). Cell 1 in somatosensory cortex encoded "running, head up" (#23) and "still,

CCW head roll” (#27); Cell 2 encoded “still, sitting” (#1). Source data are provided as a Source Data file.

Fraction of population encoding each action

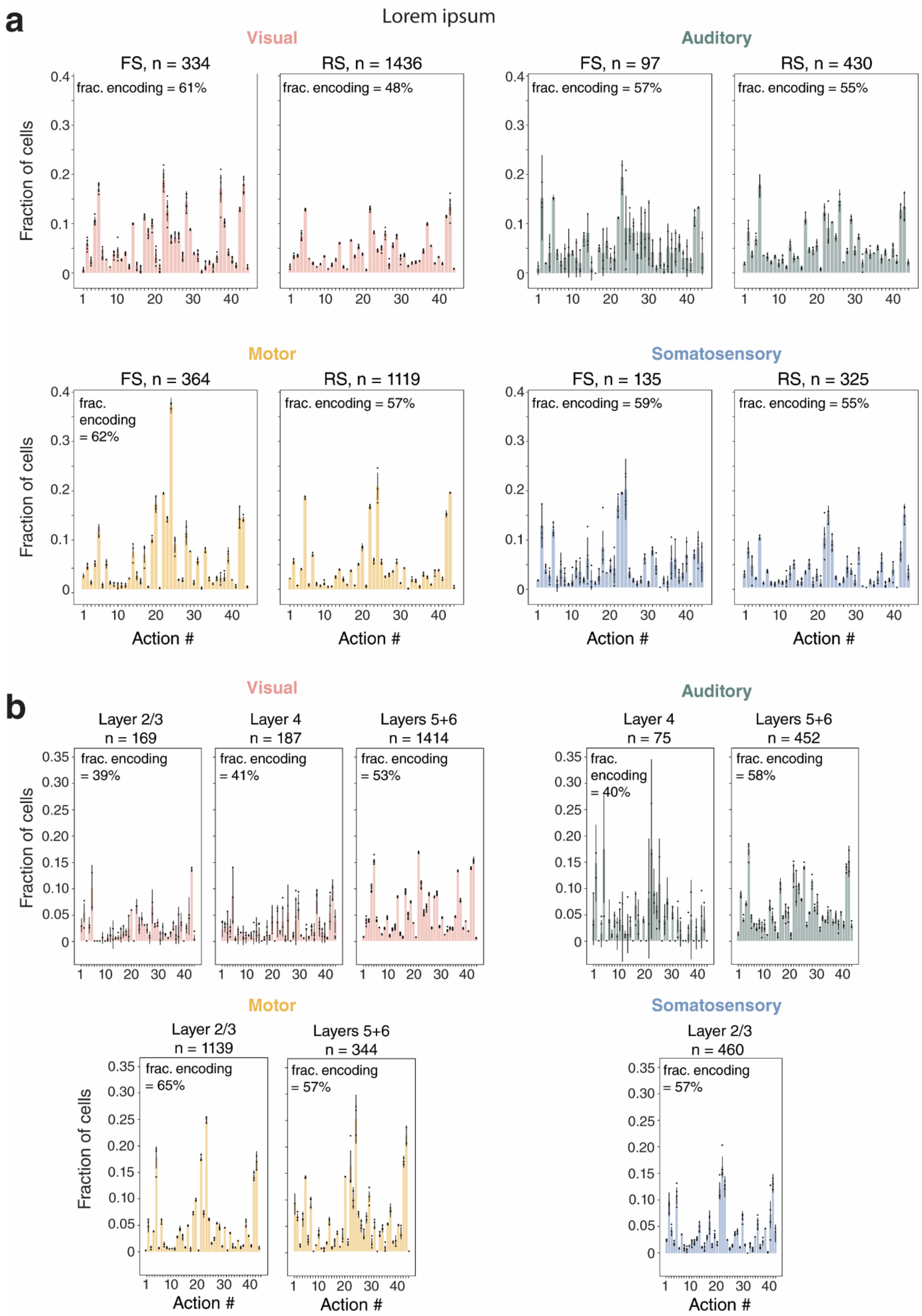

Supplementary Fig. 8: Encoding of actions across cell types and cortical layers. a, (Top left) The fraction of FS and RS neurons encoding at least one action in visual regions, with the total number of cells in each category listed above the graph, and the fraction of cells encoding at least one action at the top of each graph. (Top right) Same as for top left, but for FS and RS neurons in auditory regions. (Bottom left and right) Same as top, but for motor and somatosensory cortices, respectively. Dots denote the fraction of cells encoding an action within-animal in a given session, bars denote the means, and error bars denote ±SEM across sessions. **b**, (Top left) The total number of cells and fraction of cells encoding at least one action in layer 2/3, layer 4 and layers 5 and 6 of visual cortex. (Top right) The same for cells in layer 4 and layers 5 and 6 in auditory cortex. (Lower left) Same as above, but for layer 2/3 and layers 5 and 6 in motor cortex. (Bottom right) The fraction of cells encoding at least one action in layer 2/3 of somatosensory cortex. Dots denote the fraction of cells encoding an action within-animal in a given session, bars denote the means, and error bars denote ±SEM across sessions. Source data are provided as a Source Data file.

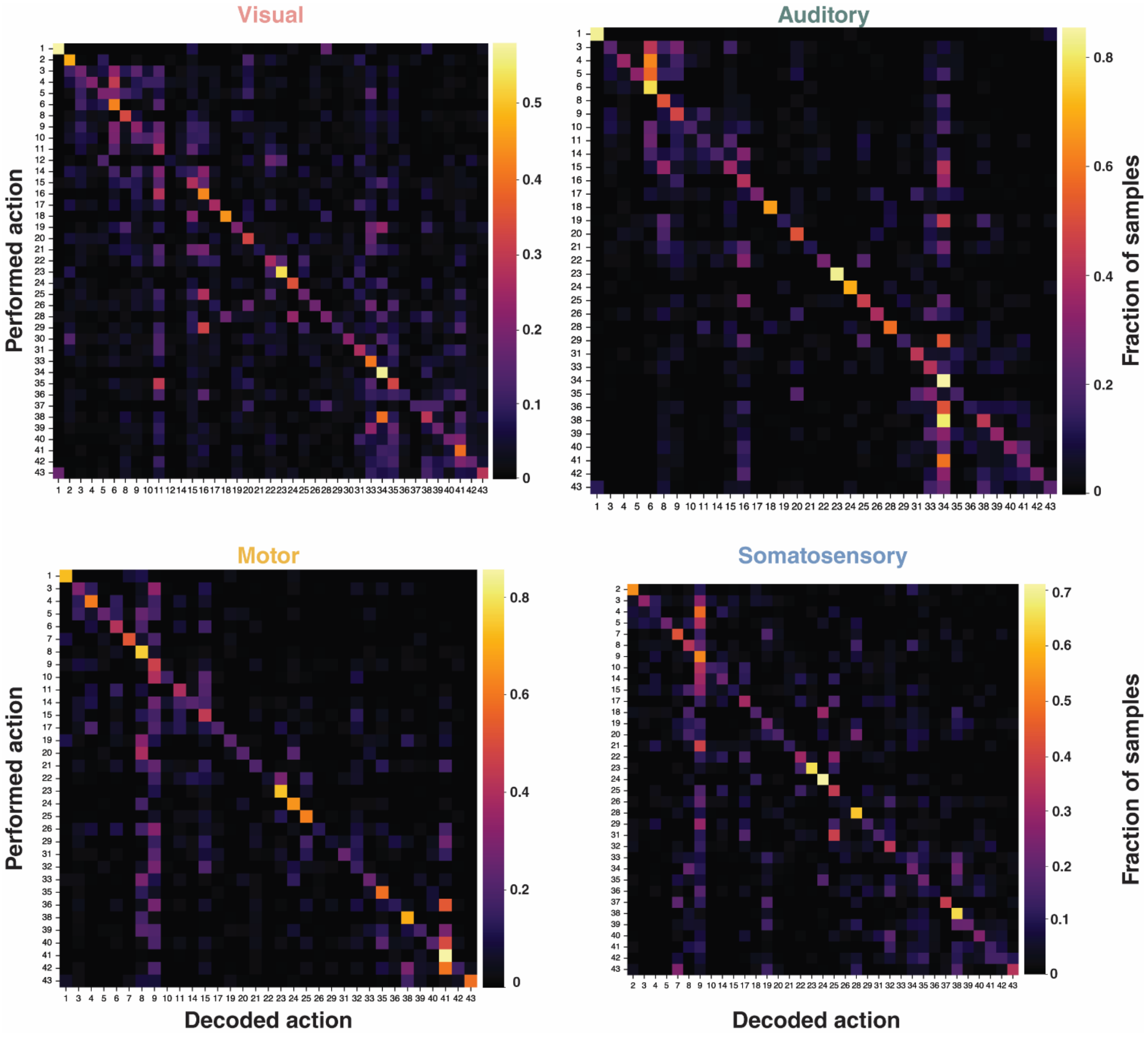

Supplementary Fig. 9: Discrimination of decoded actions in each cortical area. Normalized confusion matrices summarizing decoder performance (Methods) in classifying actions using simultaneously recorded neurons from each cortical region. The specificity of decoded actions was lower among actions sharing similar pose and movement dynamics. For example, in visual and auditory cortices, this was the case in separating Action 6 , “slow walk, head left”, from Actions 4 (”Slow walk, head left, CW head roll”) and 5 (”Still, head down left, curl left”). In motor cortex, the false positive rate was higher between Action 9 (”Walk, head bobble”) and Actions 10 (”Walk, head up left”) and 3 (”Turn left”); the same was true for actions with the head turned right (*e.g.* (#41) “Still, head turn right” was confused with (#42) “Still, curled right, head down right”). In somatosensory cortex, the lowest specificity was for Action 9 (”Walk, head bobble”), which was confused other walking-related actions such as (#4) “Slow walk, head left, CW head roll”, and (#20) “Walk, slight CW head roll, left head azimuth”. Actions with insufficient sampling were omitted; color bars indicate minimum and maximum performance; perfect performance would yield a score of 1.0. Source data are provided as a Source Data file.

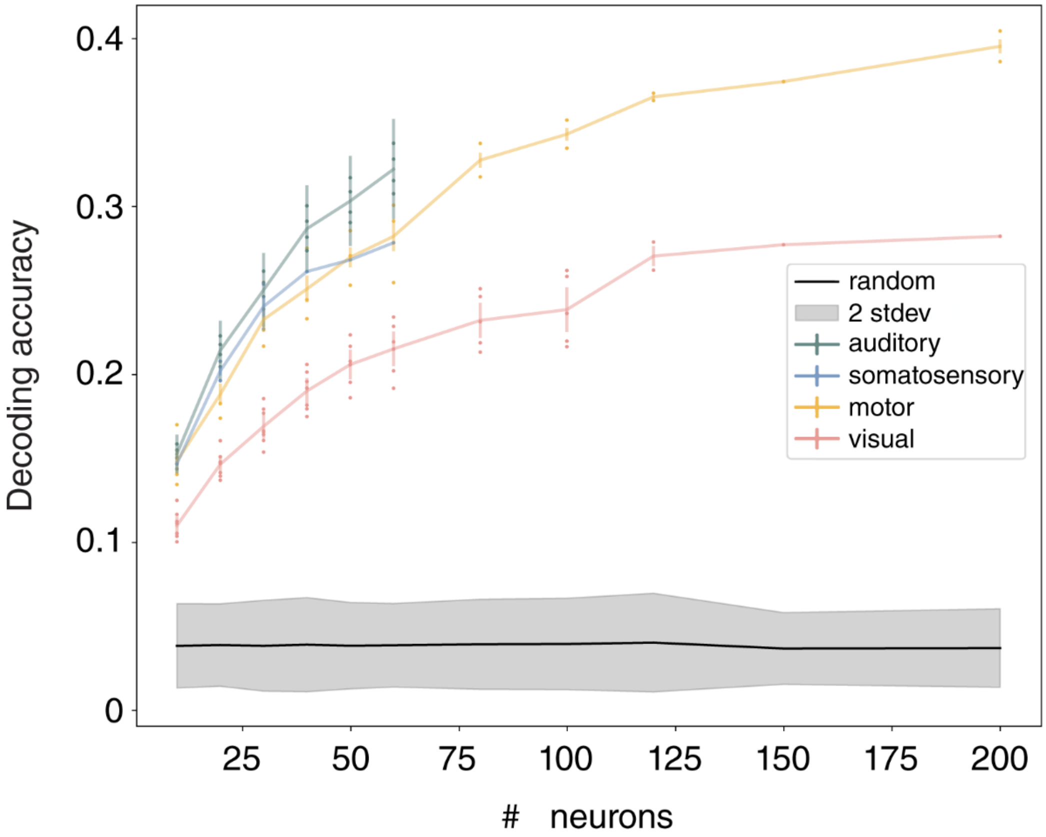

Supplementary Fig. 10: Decoding accuracy for each region increased with the number of recorded cells. Decoding accuracy was above chance levels in all cortical regions and increased when analyses included larger numbers of simultaneously recorded cells. Overall decoding accuracy was lower in visual cortex, whereas auditory, somatosensory and motor regions were comparable. The number of neurons in each analysis is on the x-axis; dots denote decoding accuracy in each session; lines denote mean accuracy; error bars denote ±SEM. Source data are provided as a Source Data file.

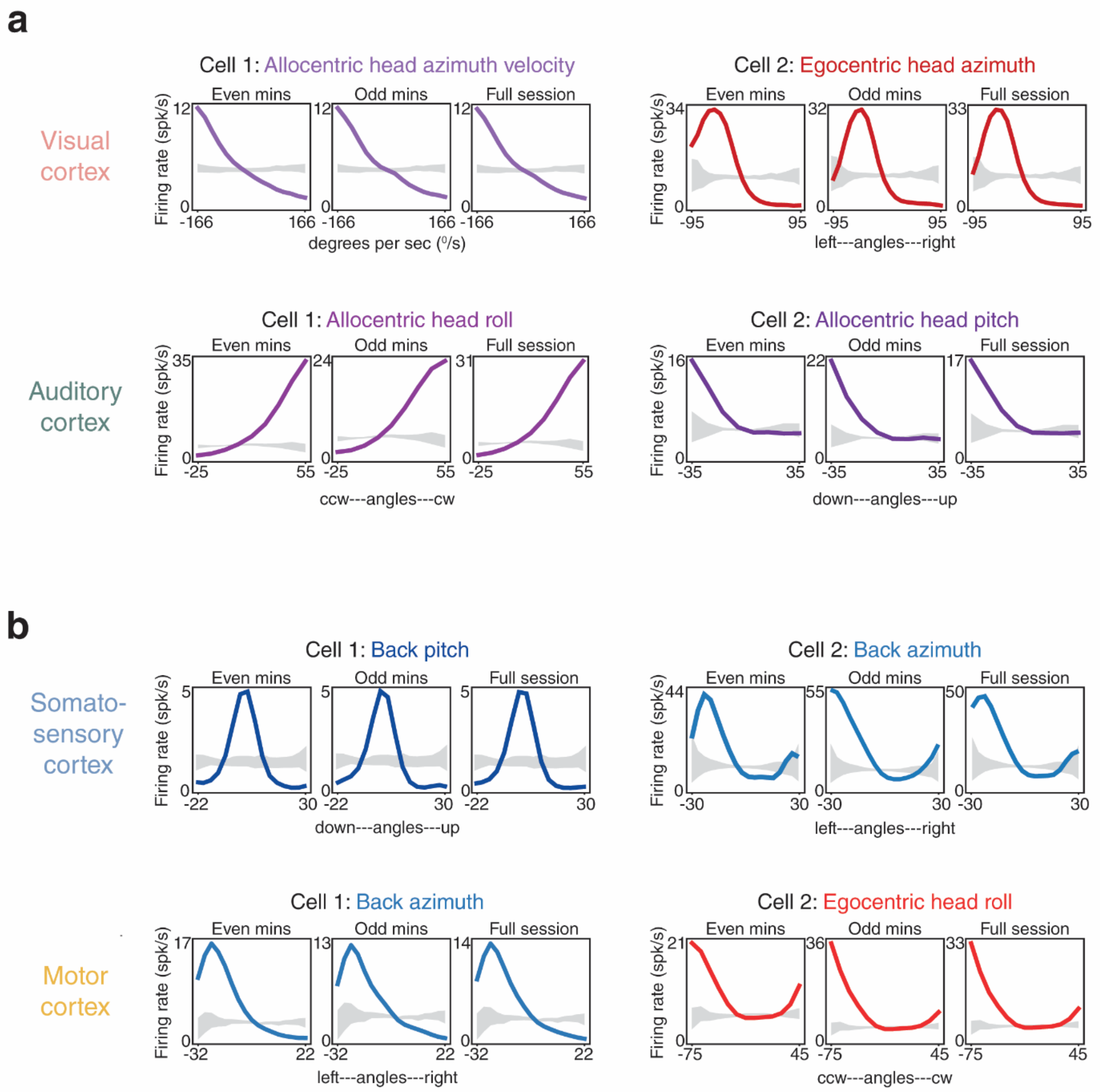

Supplementary Fig. 11: Stable posture and movement tuning curves in deep layers in each cortical area. a, (Top left) Tuning curves from a layer 6 visual cortical neuron (Cell 1) preferring leftward movement of the head in allocentric coordinates (all examples were recorded in darkness). As with Figure 2, data from even and odd minutes are shown in the left and middle, and the full session is shown to the right. Grey shading indicates the 99% CI of shuffled data. For reference to GLM analyses (in Figure 3, Supplementary Figs. 12 and 13), the pseudo-R^2 value for allocentric head pitch for Cell 1 was 0.04. (Top right) Stable tuning curves from a visual cortical neuron (layer 6) preferring left head azimuth in egocentric coordinates (*i.e.*, relative to the trunk); pseudo-R^2 of 0.13. (Lower left) A layer 6 auditory cortical neuron showing stable tuning to rightward head roll (pseudo-R^2 of 0.05), and (lower right) another L6 auditory neuron preferring downward pitch of the head in allocentric coordinates (pseudo-R^2 of 0.006). **b**, A Layer 5 somatosensory cortical neuron preferring level back pitch (top left; pseudo-R^2 of 0.07), and an S1 neuron encoding left flexion of the back (top right; pseudo-R^2 of 0.01); (Lower left) Tuning curves from L5 motor cortical neurons preferring left flexion of the back (pseudo-R^2 of 0.06), and (lower right) leftward roll of the head relative to the trunk (pseudo-R^2 of 0.01).

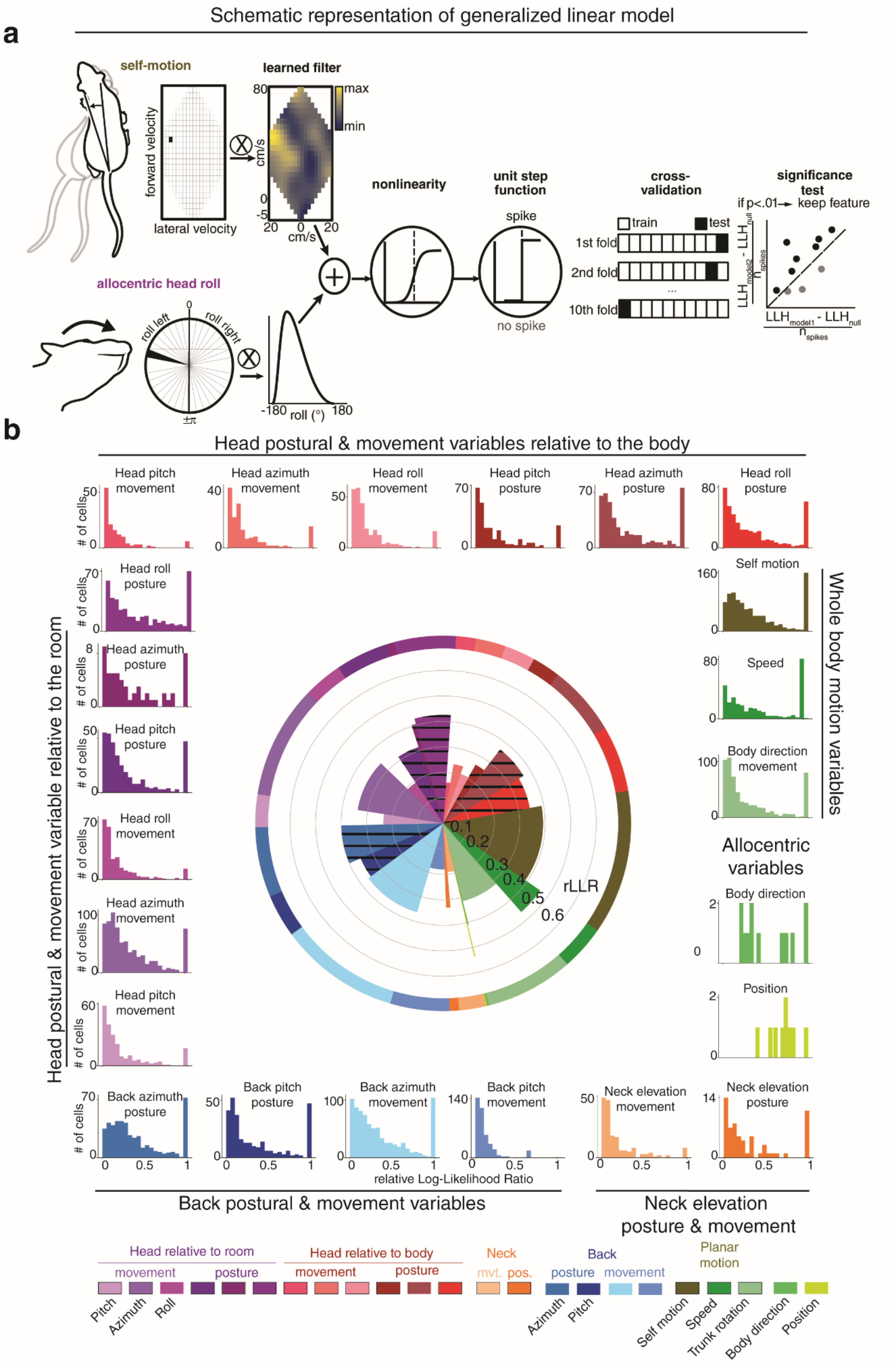
Schematic representation of generalized linear model
a
self-motion
forward velocity
lateral velocity
learned filter
80
max
min
cm/s
0
-5
20
0
20
cm/s
nonlinearity
unit step function
spike
no spike
cross-validation
train
test
1st fold
2nd fold
10th fold
significance test
if p<.01 → keep feature
allocentric head roll
roll left
roll right
0
±π
-180
roll (°)
180
b
Head postural & movement variables relative to the body
Head pitch movement
Head azimuth movement
Head roll movement
Head pitch posture
Head azimuth posture
Head roll posture
# of cells
Head postural & movement variable relative to the room
Head roll posture
Head azimuth posture
Head pitch posture
Head roll movement
Head azimuth movement
Head pitch movement
Whole body motion variables
Self motion
Speed
Body direction movement
Allocentric variables
Body direction
Position
rLLR
0.1
0.2
0.3
0.4
0.5
0.6
Back azimuth posture
Back pitch posture
Back azimuth movement
Back pitch movement
Neck elevation movement
Neck elevation posture
relative Log-Likelihood Ratio
Back postural & movement variables
Neck elevation posture & movement
Head relative to room
movement
posture
Head relative to body
movement
posture
Neck
mvt.
pos.
Back
posture
movement
Planar motion
Pitch
Azimuth
Roll
Azimuth
Pitch
Self motion
Speed
Trunk rotation
Body direction
Position

Supplementary Fig. 12: Model selection schematic and contribution to model performance of individual features. a, A toy example of the model selection procedure. A model containing one feature (self-motion, above) is augmented by the addition of a second feature (allocentric head roll, below), as the joint model significantly increases the mean cross-validated rLLR (Methods) in explaining the spiking activity of the toy neuron (far right). **b,** (Middle) Taking all non-null models from all cortical areas together, the width of each wedge indicates the relative share each variable has across all final models and length denotes the mean cross-validated rLLR of that feature across the set of such models. (Outer rim) The distribution of the cross-validated rLLRs for each feature, where the distribution of means correspond to wedge heights and the sum of each histogram normalized by the sum of all histograms corresponds to wedge widths. Source data are provided as a Source Data file. Rat illustration by Falconieri Visuals. © All Rights Reserved.

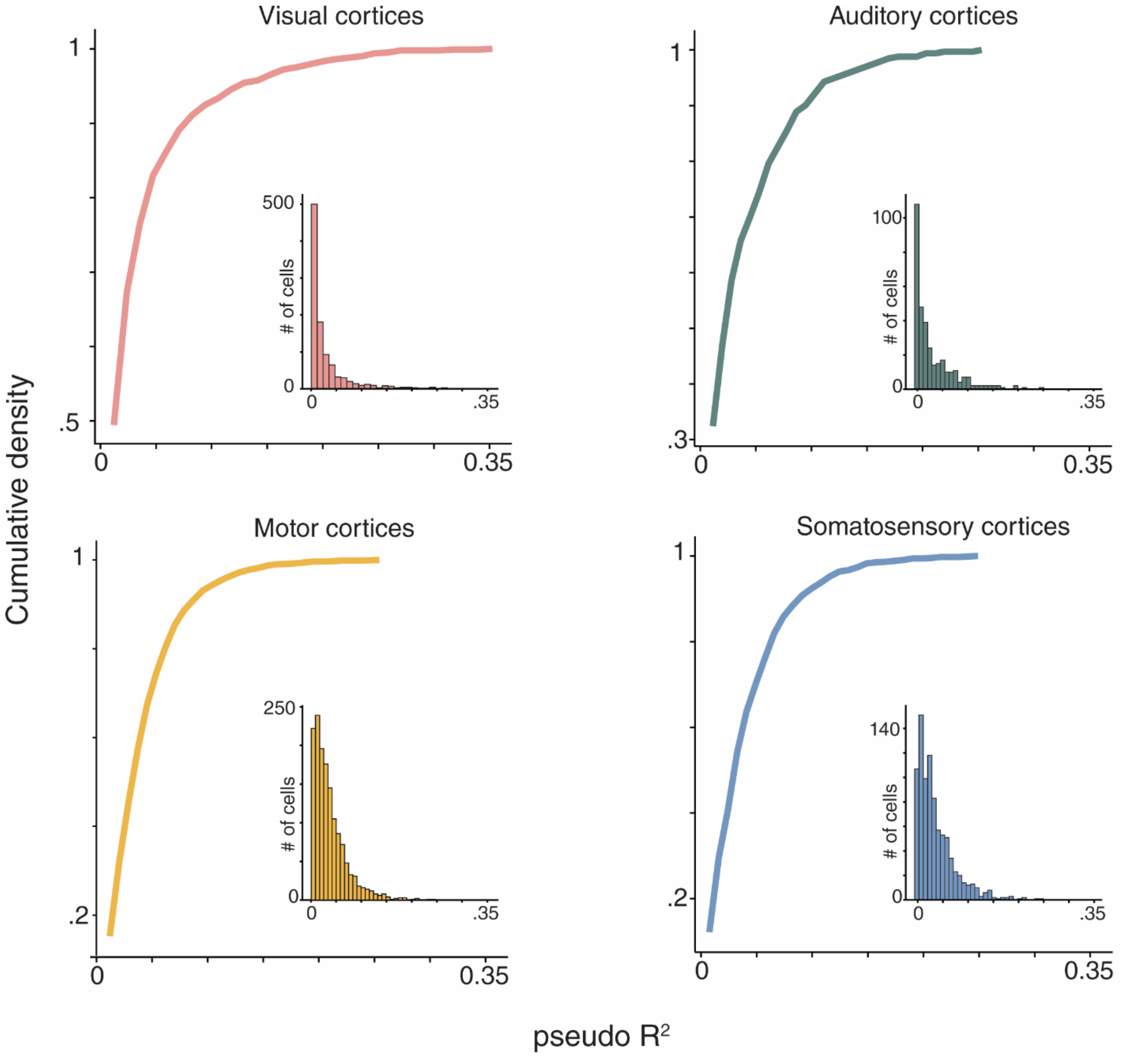

Supplementary Fig. 13: Pseudo-R^2 of generated statistical models across cortical regions. Mean out-of-sample effect size (McFadden's pseudo-R^2) distributions for models generated across the four cortical areas (visual (pink), auditory (cyan), motor (yellow) and somatosensory (blue)).

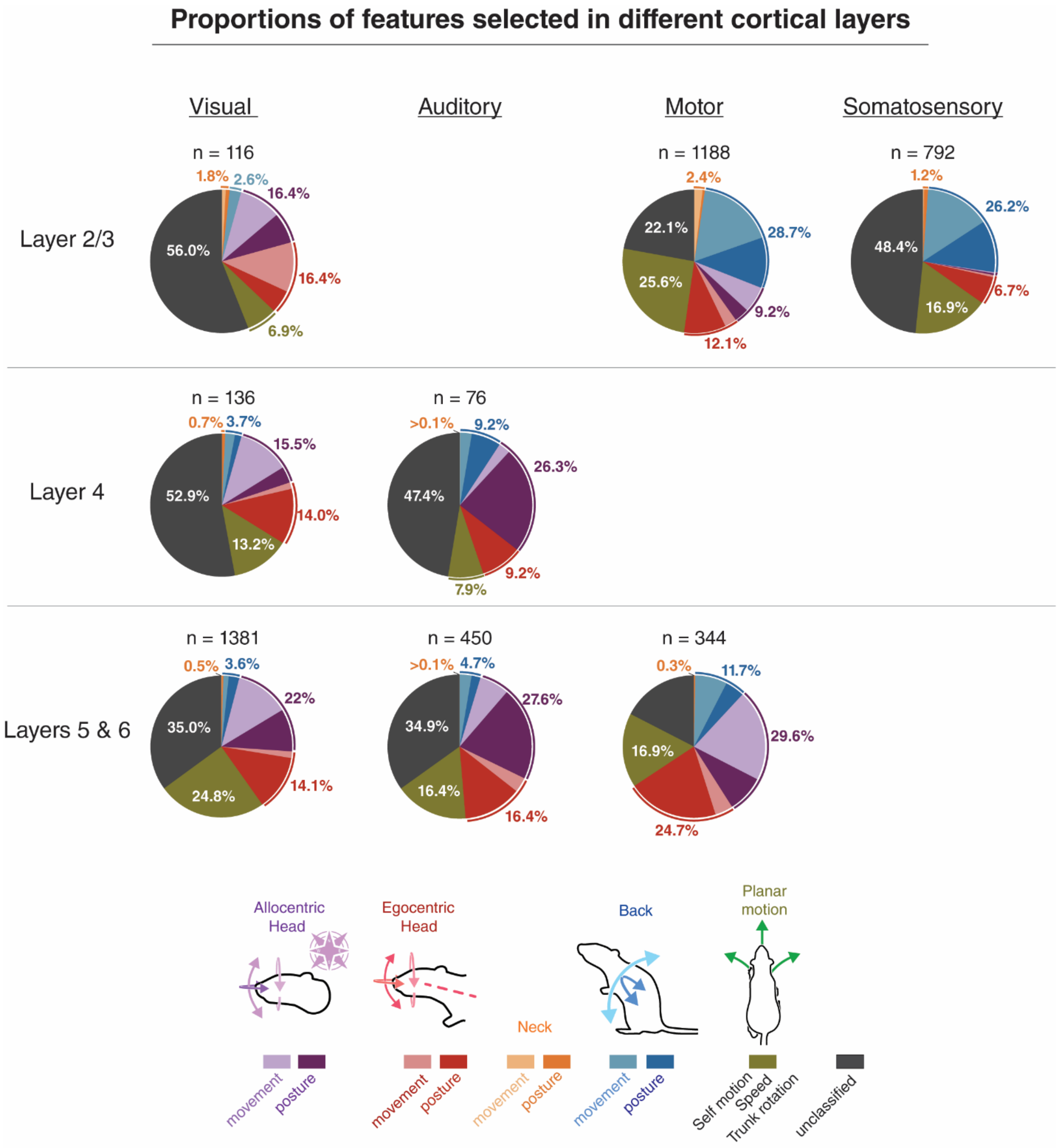

Supplementary Fig. 14: Tuning to posture, movement and self-motion across cell layers. (Top) The fraction of single units in layer 2/3 of visual, motor and somatosensory cortices for which specific behavioral features were selected as the first covariate (see color-coded legend at bottom for feature identification). (Middle) Same as the top row, but for single units recorded in layer 4 of visual and auditory cortices. (Bottom) Same, but for layers 5 and 6 in visual, auditory and motor cortices. Source data are provided as a Source Data file. Rat illustration by Falconieri Visuals. © All Rights Reserved.

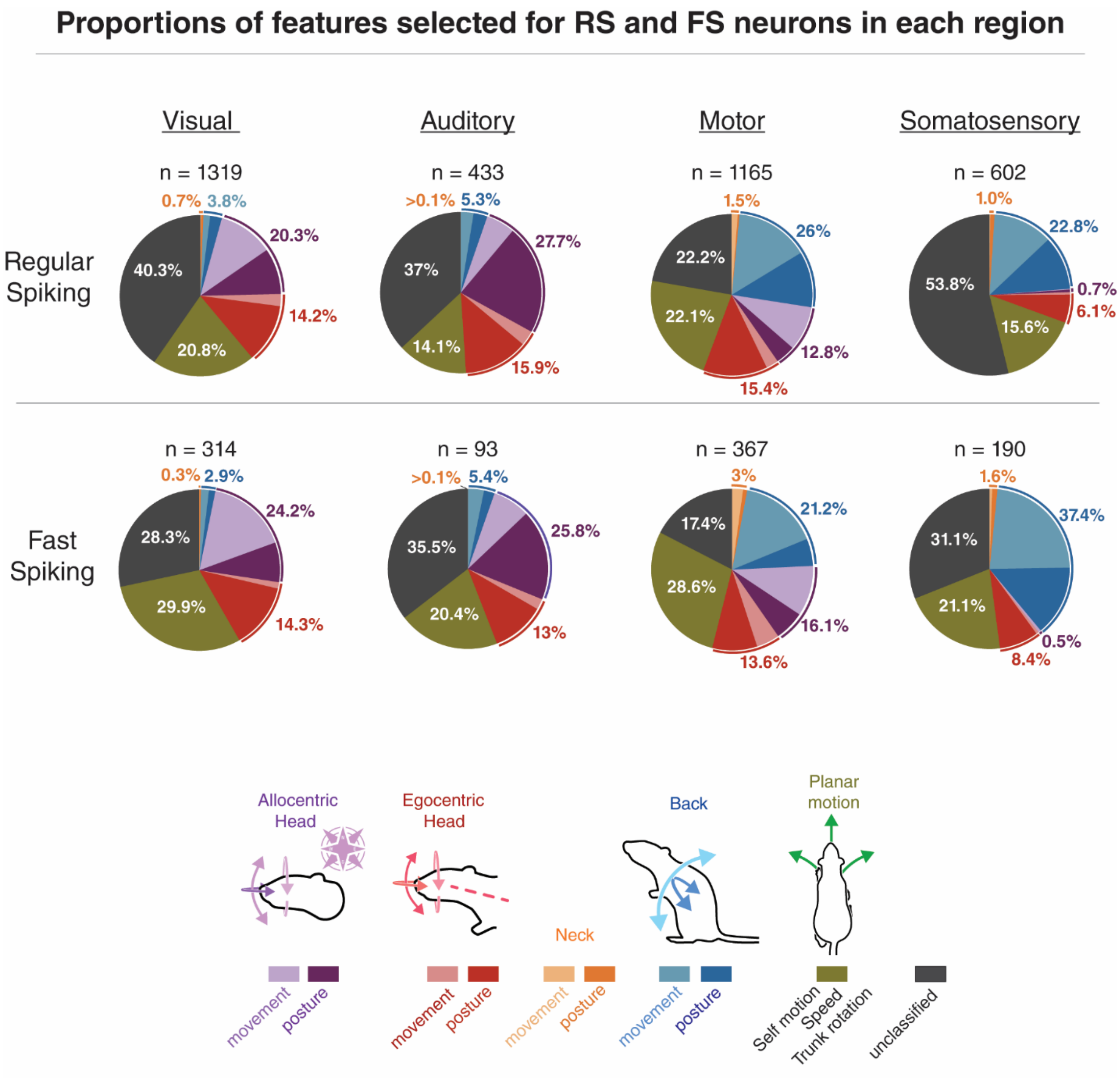

Supplementary Fig. 15: Tuning to posture, movement and self-motion in RS and FS neurons. (Top row) The fraction of regular spiking single units in visual, auditory, motor and somatosensory cortices for which a given behavioral feature was selected as the first covariate (see color-coded legend at bottom for feature identification). (Bottom row) Same as top, but for fast spiking single units in each region. Source data are provided as a Source Data file. Rat illustrations by Falconieri Visuals. © All Rights Reserved.

Proportion of tuning for all GLM-selected cells vs. those with "high" R^2

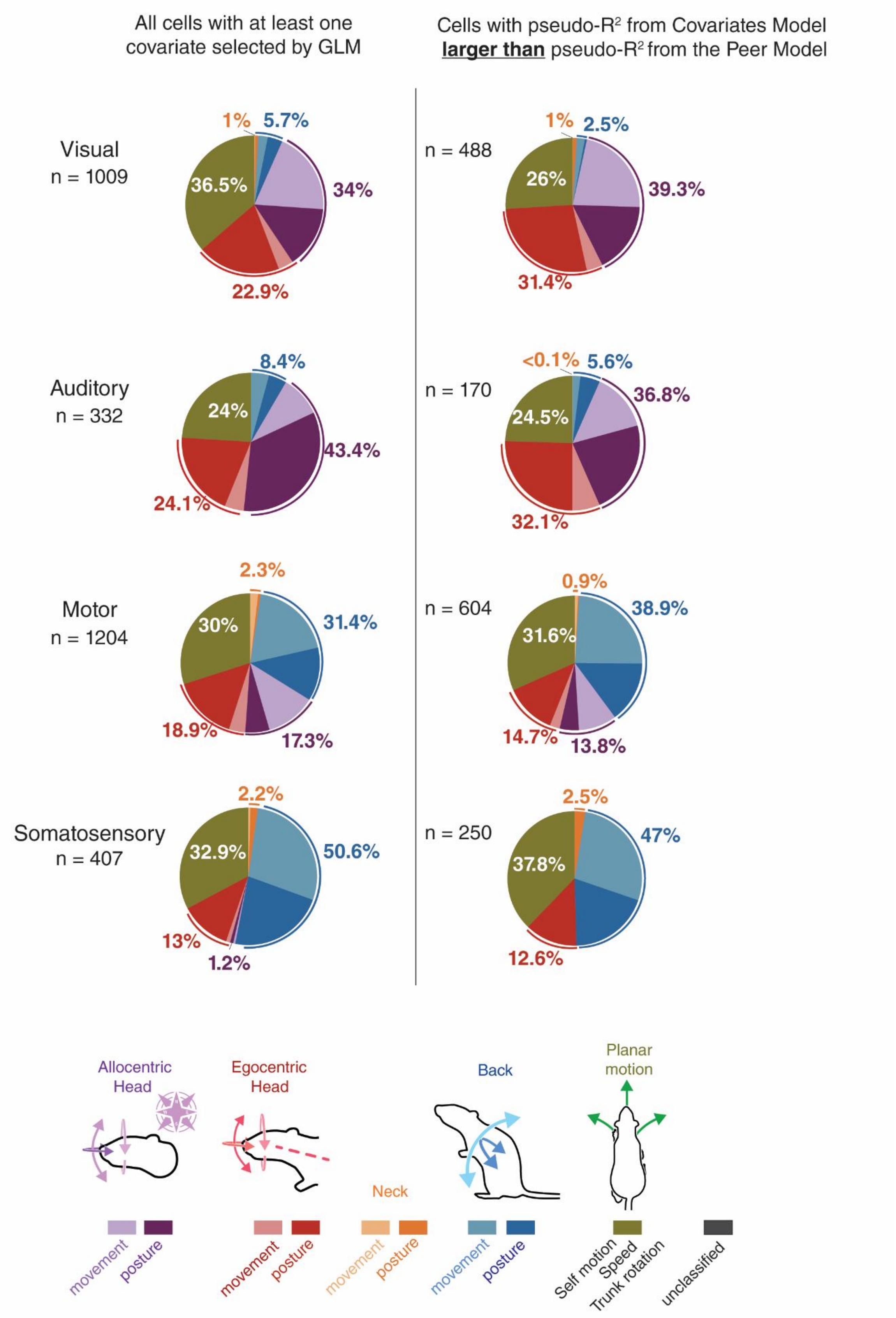

Supplementary Fig. 16: Comparison of behavioral covariates selected in GLMs using the entire dataset *versus* cells with "high" pseudo-R^2 values. To address a concern raised during review regarding the overall low pseudo-R^2 values from the GLM (referred to here as the "Covariates Model"), we compared the distribution of behavioral covariates selected from the entire dataset (Covariates Model), or only including cells with "high" pseudo-R^2 values. The threshold for "high" pseudo-R2 values was determined using a peer-prediction analysis which used the spiking activity from simultaneously recorded cells (separated by >5 recording sites on the probe) as a predicted feature[12], referred to here as the "Peer Model". Cells in the Covariates Model with pseudo-R^2 values higher than their counterparts in the Peer Model were counted as having a "high" pseudo-R^2 value. We compared the proportions of cells tuned to at least one behavioral feature from the Covariates Model (pie charts on left) *vs*. the subset of cells with "high" pseudo-R^2 values (pie charts on right), and found that the regional differences in encoding properties (*e.g.* predominant encoding of allocentric and egocentric head features in visual and auditory cortices) were upheld when only considering the cells with "high" pseudo-R^2 values. Source data are provided as a Source Data file. Rat illustrations by Falconieri Visuals. © All Rights Reserved.

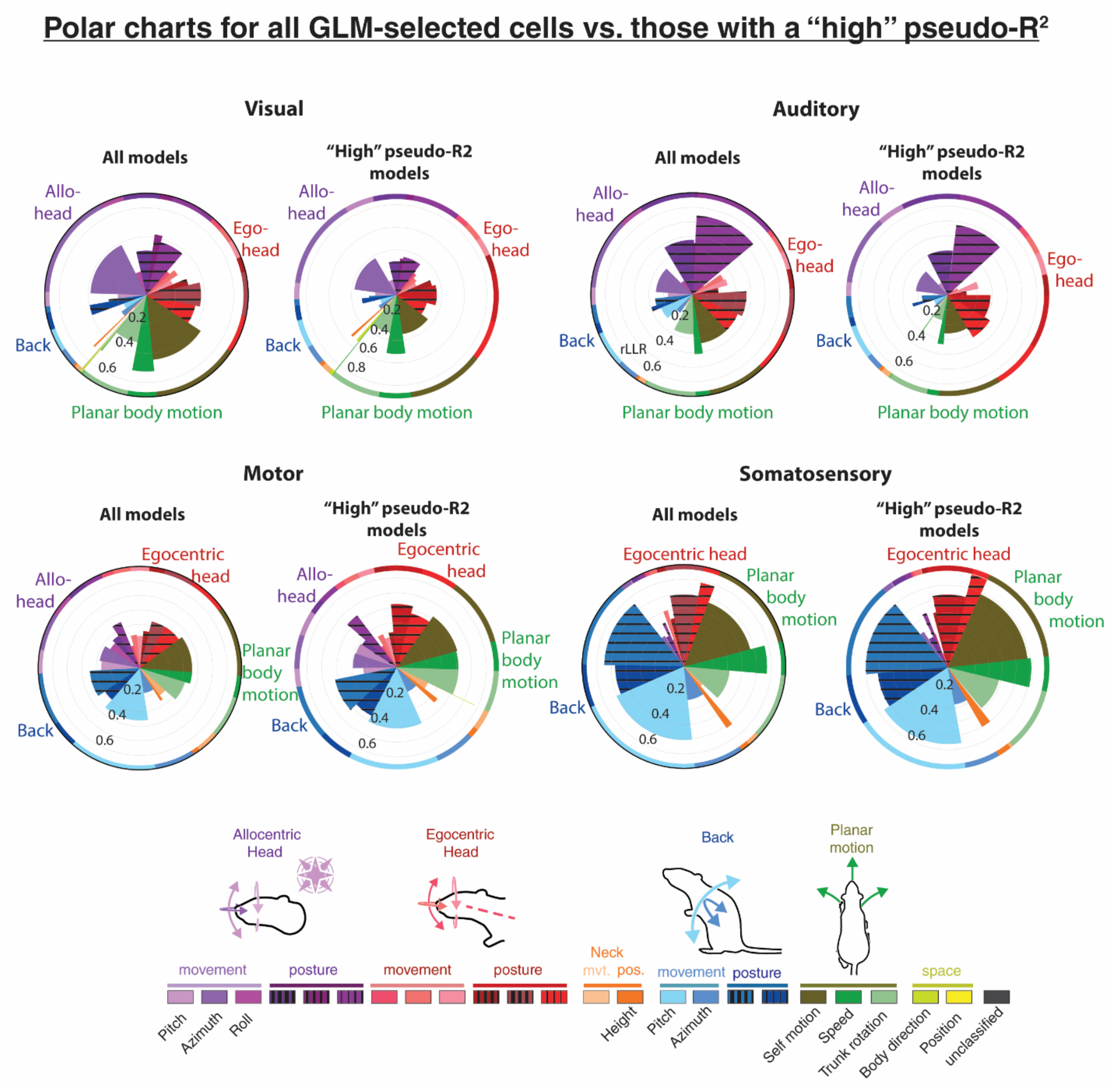

Supplementary Fig. 17: Polar charts comparing the proportions of tuned cells and rLLR values for all GLM-selected cells *versus* those with "high" pseudo-R^2 values. Polar charts from each cortical region denoting the relative importance of individual covariates included in the full model ("All models", left) *versus* those from cells with pseudo-R^2 values higher than their Peer Model ("High" pseudo-R2 models, right; same "Peer Model" analysis as Supplementary Fig. 16). "Unclassified" cells are excluded in the polar charts; wedge length denotes the mean cross-validated rLLR (Methods) of each covariate across the set of models it was included in. As in Figure 3, the width of each wedge reflects the fraction of times that feature was selected among the other covariates. Wedge length and width are independent of each other (*i.e.* the area does not reflect effect size). Wedge widths and lengths in each region were largely similar for full models and models only including cells with "high" pseudo-R^2 values. Source data are provided as a Source Data file. Rat illustrations by Falconieri Visuals. © All Rights Reserved.

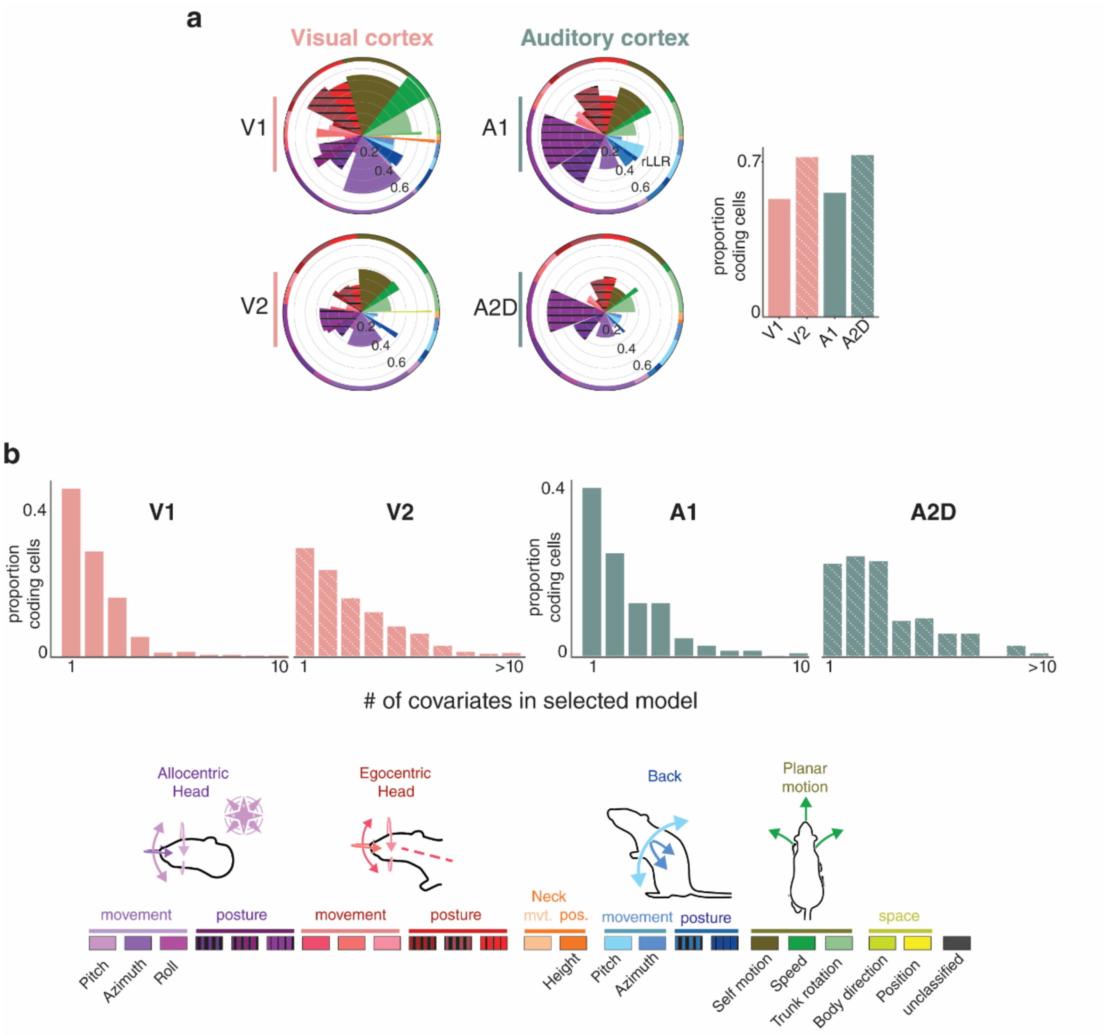

Supplementary Fig. 18: Comparison of tuning properties across primary and secondary sensory regions. a, (Left) Relative variable share and mean cross-validated rLLR of each feature across models for V1 (top) and V2 (V2M, V2L) (below). (Middle) same as (left), but for A1 and A2D. (Right) The proportion of single units encoding at least one behavioral feature in visual (pink) and auditory (cyan) regions. **b**, The proportion of tuned neurons statistically linked to one or any larger number of behavioral covariates (from left to right: V1, V2, A1, A2D). Source data are provided as a Source Data file. Rat illustrations by Falconieri Visuals. © All Rights Reserved.

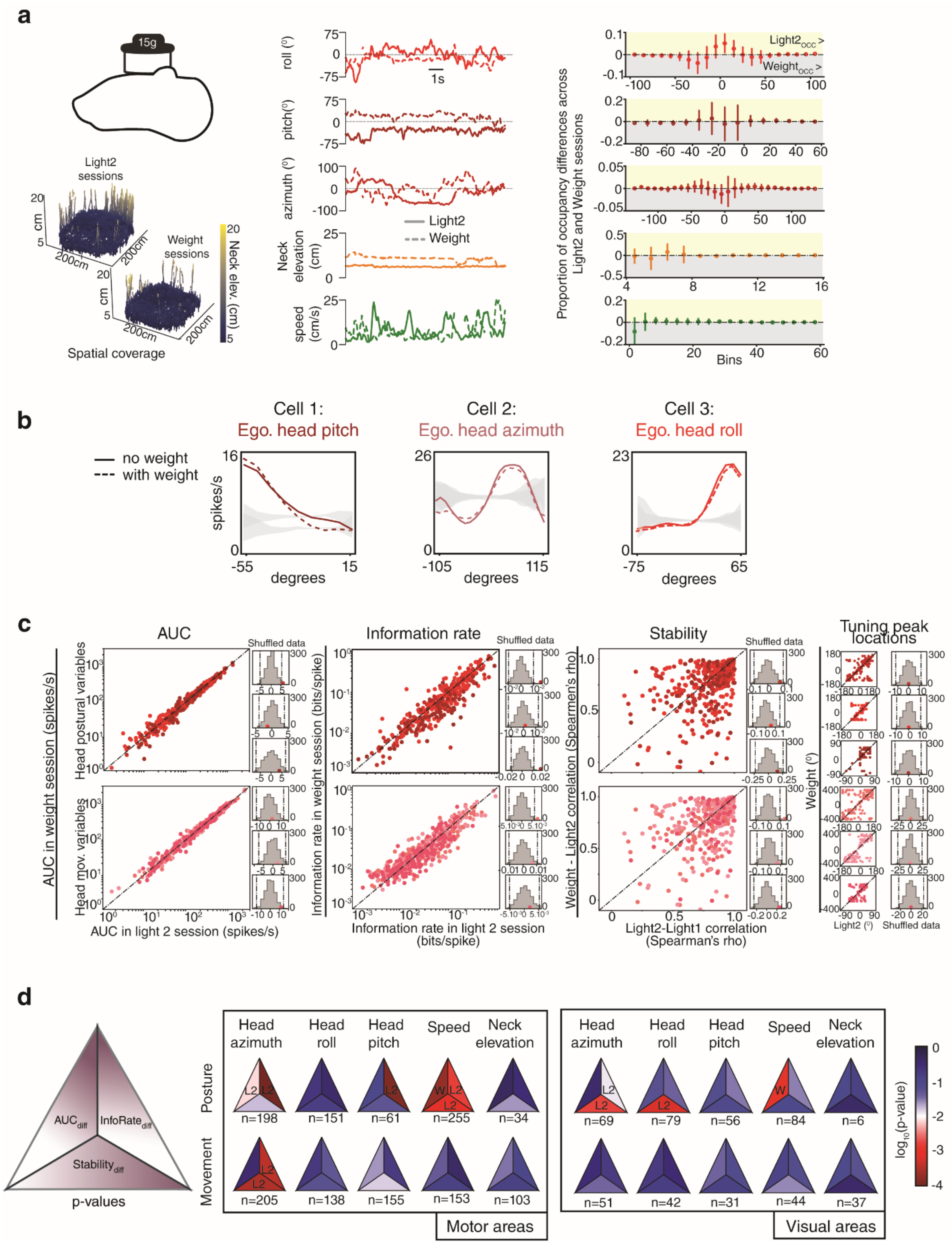

a
15g
Light2 sessions
Weight sessions
Neck elev. (cm)
Spatial coverage
roll (°)
pitch(°)
azimuth (°)
Neck elevation (cm)
speed (cm/s)
1s
Light2
Weight
Proportion of occupancy differences across Light2 and Weight sessions
Light2occ >
Weightocc >
Bins
b
no weight
with weight
Cell 1:
Ego. head pitch
Cell 2:
Ego. head azimuth
Cell 3:
Ego. head roll
spikes/s
degrees
c
AUC
Information rate
Stability
Tuning peak locations
Shuffled data
AUC in weight session (spikes/s)
Head postural variables
Head mov. variables
AUC in light 2 session (spikes/s)
Information rate in weight session (bits/spike)
Information rate in light 2 session (bits/spike)
Weight - Light2 correlation (Spearmen's rho)
Light2-Light1 correlation (Spearman's rho)
Weight (°)
Light2 (°)
d
AUCdiff
InfoRatediff
Stabilitydiff
p-values
Head azimuth
Head roll
Head pitch
Speed
Neck elevation
Posture
Movement
n=198
n=151
n=61
n=255
n=34
n=205
n=138
n=155
n=153
n=103
Motor areas
n=69
n=79
n=56
n=84
n=6
n=51
n=42
n=31
n=44
n=37
Visual areas
log10(p-value)

Supplementary Fig. 19: Effects of additional head-mounted weight on behavioral tuning. a, (Upper left) Small, cuboid copper alloy (15 g) relative to the chronic implant on a rat's head. (Lower left) Differences in the vertical behavior between four left hemisphere (LH-implanted) animals across light/weight sessions. (Middle) Example traces for five features (head azimuth, head roll, head pitch, neck elevation and speed) across light/weight conditions of one LH rat (#26504). (Right) 99% CIs (mean ± 2.58 SEM) of occupancy differences for the same five features across light/weight conditions calculated over all LH and RH rats (n = 6)**. b**, Representative examples of cells with similar tuning curves for pitch, azimuth and roll of the head with and without the 15 g head weight; the 99% CI of shuffled data are shown in grey. **c**, Quantitative comparison of tuning features for motor cortex units with stable baseline firing rates (Methods) across sessions with (”weight sessions“) or without (”Light 2“) added head weight. (Left) The area under tuning curves (AUC) for head posture and movement features; (middle left) the information rate for each feature (calculated in bits/spike for each cell[101]), (middle right) the stability and (right) tuning peak locations across sessions. The means of the distributions for the three head postural/movement features (red dots) are shown relative to their respective shuffled distributions (grey). **d**, (Left) Schematic showing which p-value is presented in which part of the triangle; "diff” is for comparison between light2 and weight sessions. (Middle) Statistical significance (color) and direction (session label in triangle) of the difference relative to its shuffled distribution for all tuned single units with stable baselines in motor cortex. (Right) For comparison, the same tests were run on single units in visual areas. Source data are provided as a Source Data file. Rat illustration by Falconieri Visuals. © All Rights Reserved.

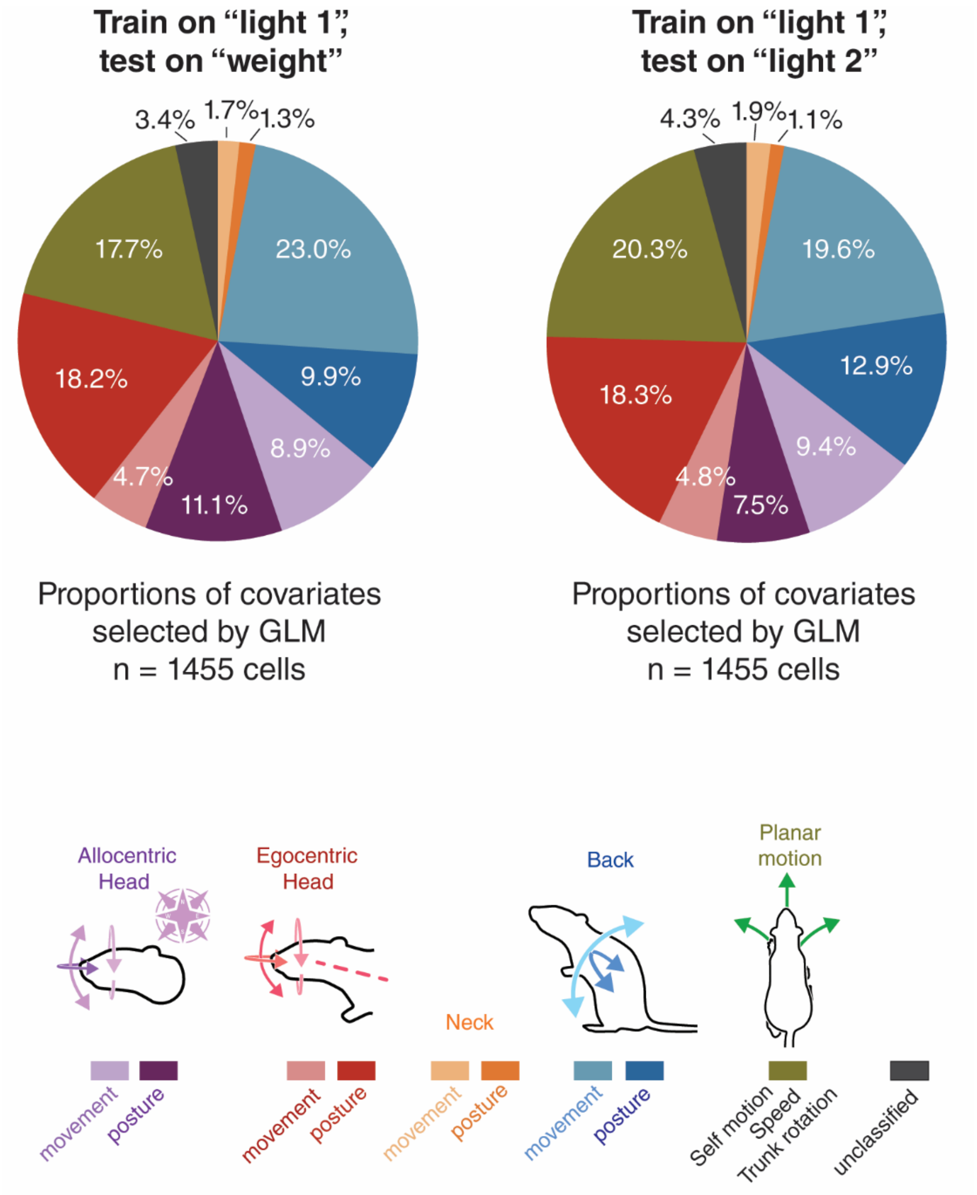

Supplementary Fig. 20: Comparison of behavioral covariates selected across light1-weight and light1-light2 sessions. The proportions of behavioral covariates selected by GLMs trained on data recorded during the first weight-free session (light 1) and tested on recordings with a 15 g weight on the head (left pie chart) or a second weight-free (light 2, right) recording session. The proportions of cells encoding ego- and allocentric head features were similar across weight and weight-free conditions, differing from 0.1% (for egocentric head posture) to 3.6% (for allocentric head posture). Source data are provided as a Source Data file. Rat illustration by Falconieri Visuals. © All Rights Reserved.

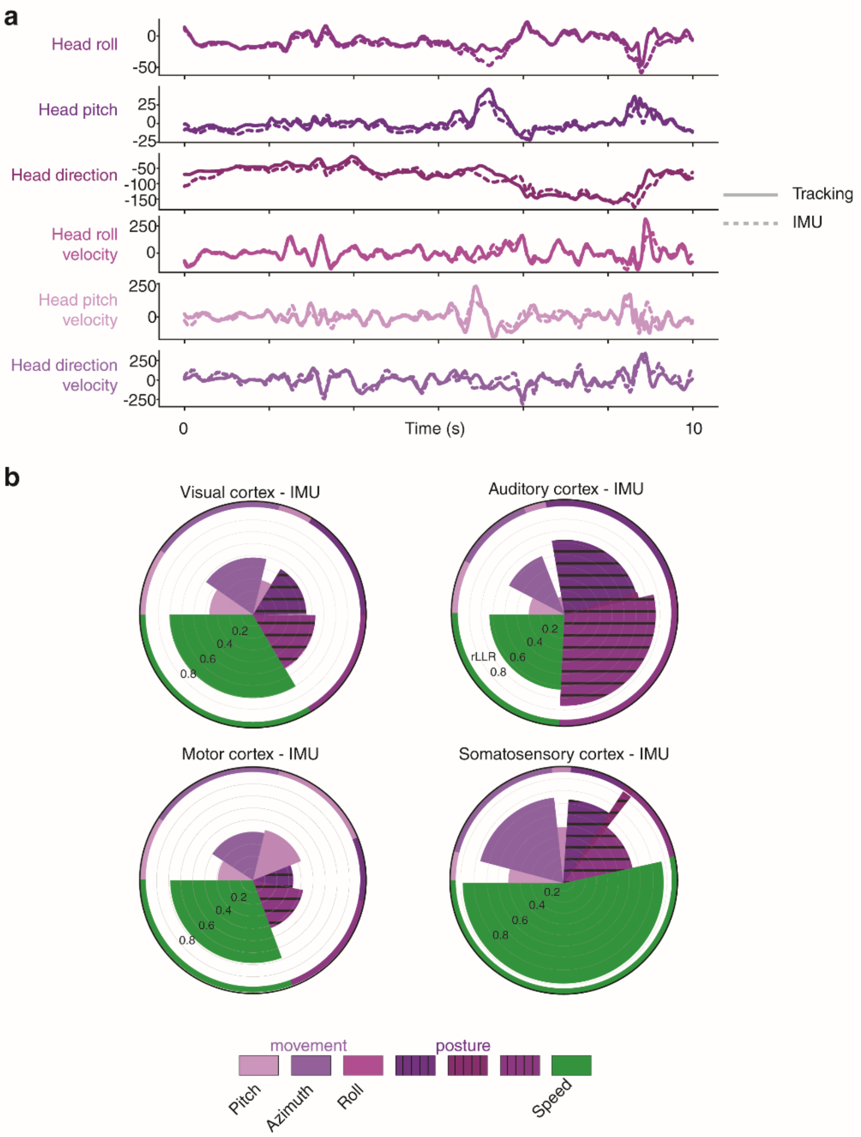

Supplementary Fig. 21: Differences between optical tracking and IMU-generated Euler angles in explaining spiking activity of individual neurons. a, A behavioral recording segment depicting dynamics of six features (allocentric head roll, -head pitch, -head direction, and their velocities), as defined by optical tracking (solid line) or the IMU (dashed line) in one right hemisphere-sampled rat (#26525). **b,** The relative importance of individual covariates (features in **a** and speed) in the data as in Fig. 3. (Top) Polar plots represent GLM covariate prevalence from one right hemisphere animal (#26525) using IMU-generated Euler angles for visual cells (left) and auditory cells (right). (Bottom) Polar plots represent GLM covariate prevalence from one left hemisphere animal (#26472) using IMU-generated Euler angles for motor cells (left) and somatosensory cells (right). Source data are provided as a Source Data file.

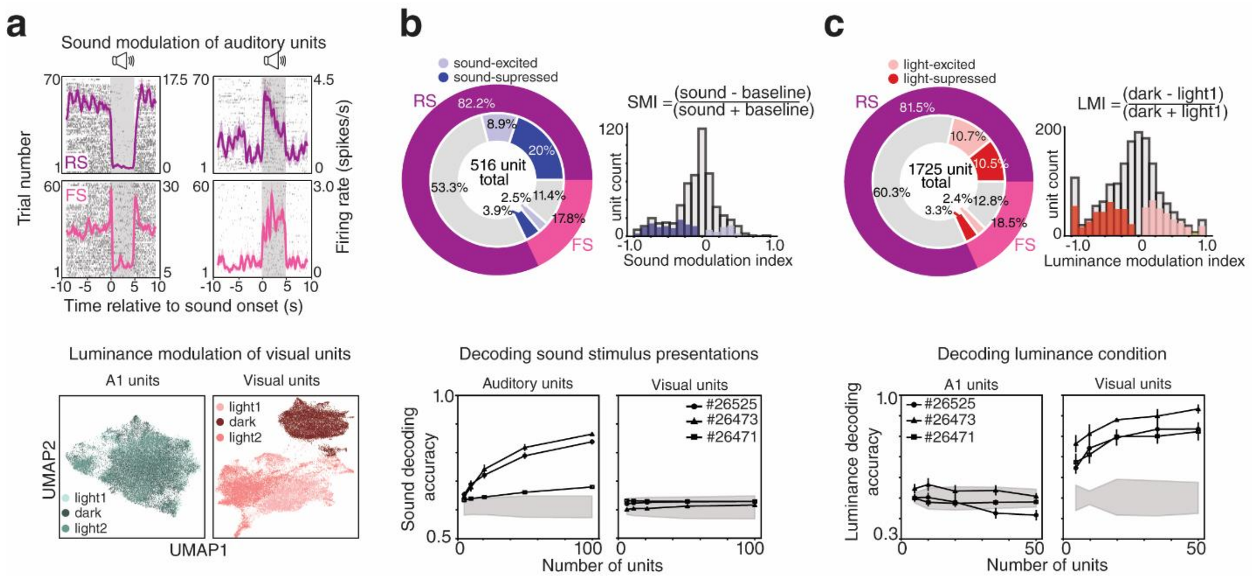

Supplementary Fig. 22: Modality specificity and decoding using auditory and visual cortical units. a, (Top) Spike rasters (grey) and peri-event time histograms (PETHs) of sound-suppressed (left) and sound-excited (right) auditory cortical single units (purple line and shading depict trial averaged firing rate ±3 SEM for regular spiking (RS) units; the same for fast spiking (FS) units is shown in magenta; sound stimulation in grey shading). (Bottom) Non-linear embedding of A1 population vector activity from light and dark recording sessions for an example rat (#26525); (right) same but for visual cortical units. **b**, (Top left) Proportions of significantly sound-suppressed (dark blue) and sound-excited (light blue) RS and FS auditory units. (Top right) The sound modulation index distribution for all (grey), significantly suppressed (dark blue) and significantly excited (light blue) auditory units. (Bottom) Decoding of sound stimulation with auditory (left) and visual (right) single units (symbols and vertical lines show mean decoding accuracy ±3 SEM for each rat; shaded area is 99% of the shuffled distribution); n = 516 single units. **c**, (Top left) Proportions of significantly dark-suppressed (dark red) and dark-excited (light red) RS and FS units in visual cortices. (Top right) The luminance modulation index distribution for all (grey), significantly dark-suppressed (dark red) and significantly dark-excited (light red) visual units. (Bottom) Decoding of luminance condition with A1 (left) and visual cortex (right) single units (symbols and vertical lines same as in **b**); n = 1725 single units. Source data are provided as a Source Data file.

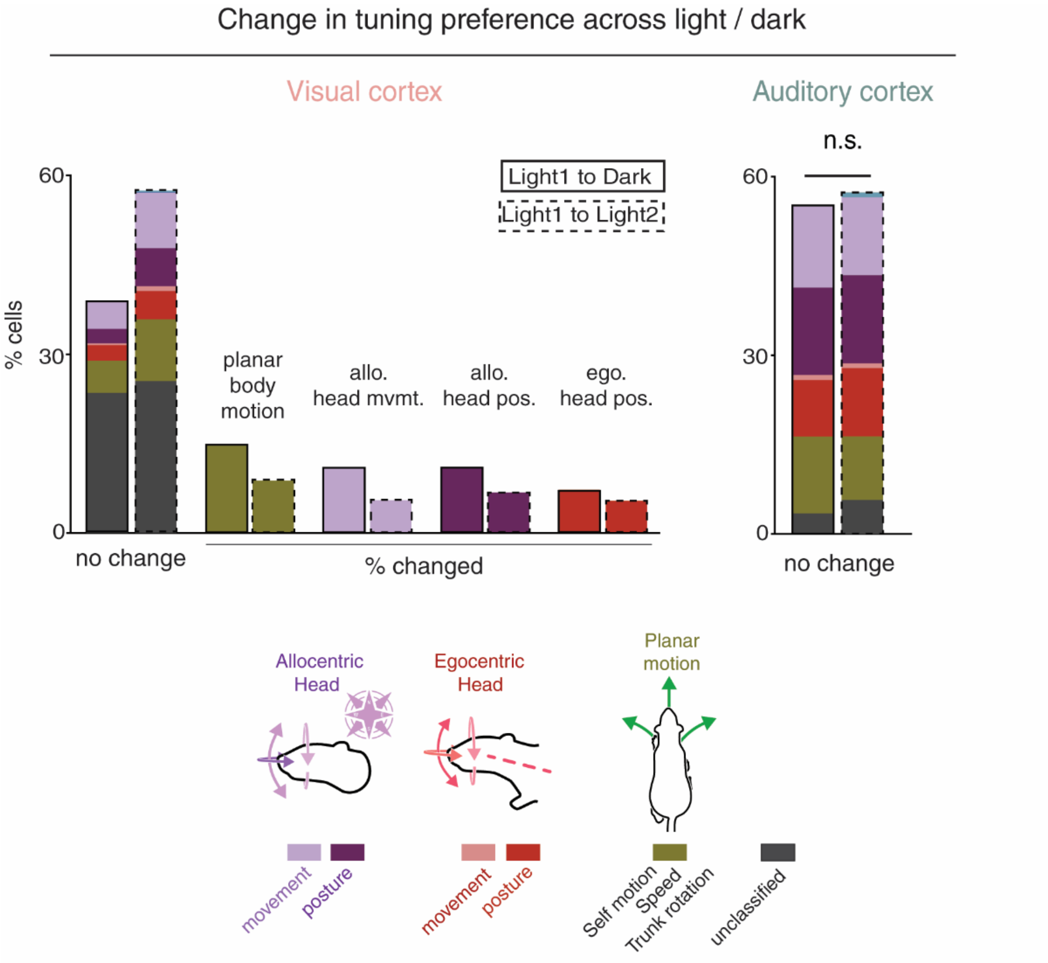

Supplementary Fig. 23: Instability of movement and pose correlates in visual, but not auditory cortices across light and dark conditions. GLM-selected behavioral covariates in visual (left), but not auditory (right), cortex were less stable across light-dark (solid outline) than light-light (dashed outline) recording sessions. Cells encoding planar body motion ($p = 2^{-15}$), allocentric head movement ($p = 8^{-7}$), allocentric ($p = 1.7^{-8}$) and egocentric head posture ($p = 1^{-11}$) were less stable across light-dark than light-light recording conditions (two sample Z-test for proportions (2-sided)). Source data are provided as a Source Data file. Rat illustrations by Falconieri Visuals. © All Rights Reserved.

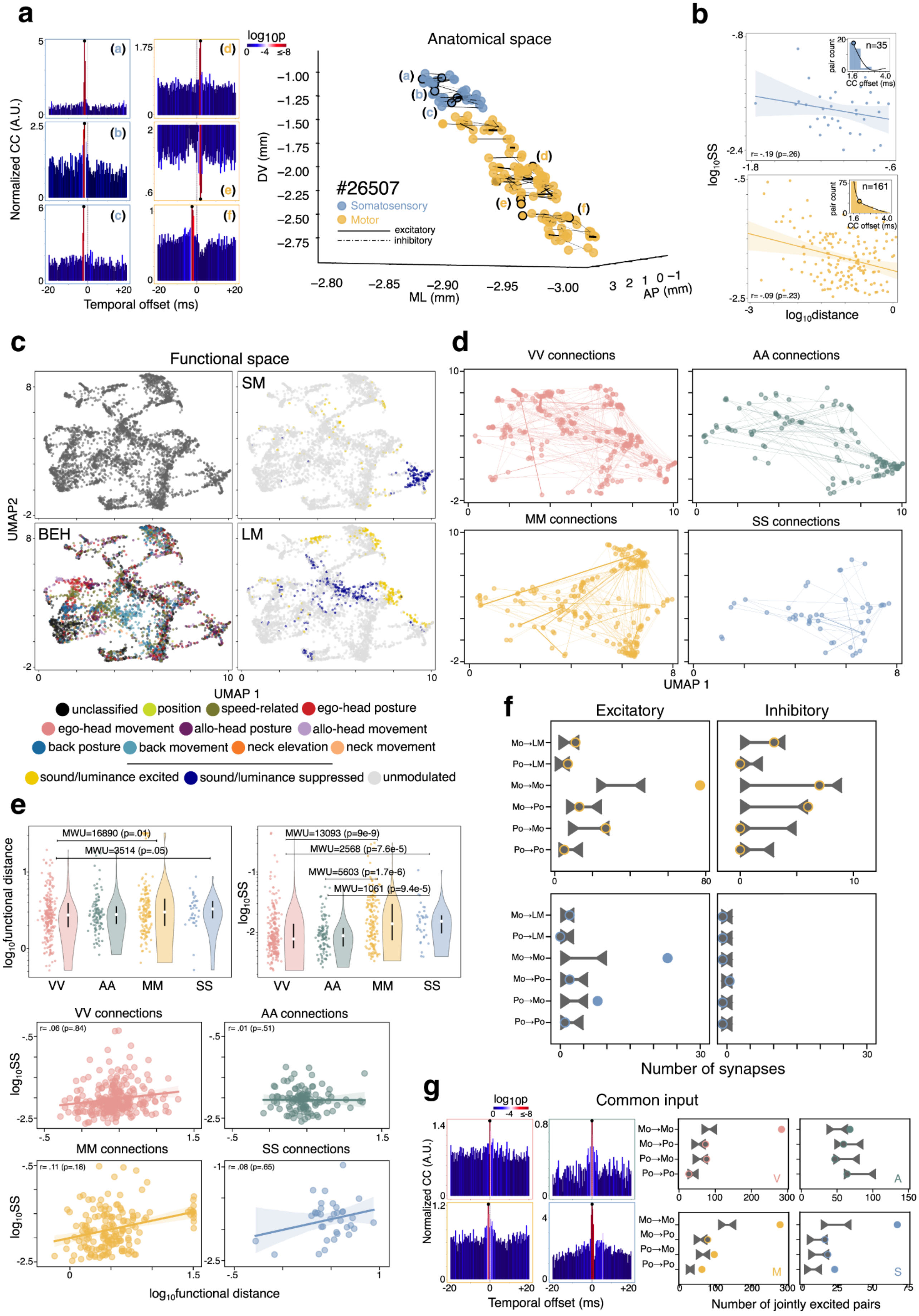
a
Normalized CC (A.U.)
Temporal offset (ms)
log10p
Anatomical space
#26507
Somatosensory
Motor
excitatory
inhibitory
DV (mm)
ML (mm)
AP (mm)
b
log10SS
log10distance
n=35
n=161
pair count
CC offset (ms)
r= -.19 (p=.26)
r= -.09 (p=.23)
c
Functional space
SM
BEH
LM
UMAP2
UMAP 1
unclassified
position
speed-related
ego-head posture
ego-head movement
allo-head posture
allo-head movement
back posture
back movement
neck elevation
neck movement
sound/luminance excited
sound/luminance suppressed
unmodulated
d
VV connections
AA connections
MM connections
SS connections
UMAP 1
e
log10functional distance
MWU=16890 (p=.01)
MWU=3514 (p=.05)
log10SS
MWU=13093 (p=9e-9)
MWU=2568 (p=7.6e-5)
MWU=5603 (p=1.7e-6)
MWU=1061 (p=9.4e-5)
VV
AA
MM
SS
VV connections
AA connections
MM connections
SS connections
r= .06 (p=.84)
r= .01 (p=.51)
r= .11 (p=.18)
r= .08 (p=.65)
log10functional distance
f
Excitatory
Inhibitory
Mo→LM
Po→LM
Mo→Mo
Mo→Po
Po→Mo
Po→Po
Number of synapses
g
Common input
Normalized CC (A.U.)
Temporal offset (ms)
Number of jointly excited pairs
V
A
M
S

Supplementary Fig. 24: Properties of synaptically coupled neuronal pairs. a, (Left) Three temporal spiking cross-correlograms from S1 (a-c) and M1 cortices (d-f); significance threshold was set at the 99.9999% or 0.0001% of the cumulative Poisson distribution for excitatory or inhibitory connections, respectively; P-value calculations described in detail in Methods sub-section "Functional connectivity". (Right) all putative synaptic connections including examples (a-f) from one rat's (#26507) S1 (blue) and M1(yellow) cortices in anatomical space; line widths indicate synaptic strength (SS). **b**, (Top) Log-log relationship between anatomical distance and SS for putative connections in S1, (inset) distribution and median (colored circle) of cross-correlogram peaks/troughs; (bottom) same, for M1. There was no relation between SS and anatomical distance in either region (in S1, two-sided r = -0.19, p = 0.26; in M1, two-sided r = -0.09, p = 0.23). Lines in the scatter points denote the least squares regression fit, shaded areas denote 95% CI. **c**, (Top left) All recorded cells projected on the low-dimensional covariate embedding; (bottom left) same as (top left), with cells color coded by the covariate providing the best fit to their spiking. (Top right) Same as (top left), colors indicate sound modulation category. (Bottom right) Same as (top left), colored by luminance modulation. **d**, (Top left) All visual-visual synaptic connections plotted in functional space (line width denotes SS). (Bottom left) Same as (top left), for motor-motor synaptic connections. (Top right) Same as (top left) for auditory-auditory connections; (bottom right) same, for somatosensory-somatosensory connections. **e**, (Top left) Functional distance distributions for all synaptically coupled pairs in each area (between 247 cells in visual cortex; 107 in auditory; 181 in motor; 35 in somatosensory). (Top right) Same as (top left), only for SS (circles denote the median, error bars the 25th and 75th percentiles of the distributions). (Bottom) Correlations between functional distance and synaptic strength in each area (all plots visualized on log or log-log scales; statistics performed on original data using a two-sided Mann–Whitney U (MWU) test; lines in scatter points denote least squares regression fit, shaded areas denote the 95% CI.). **f**, Shuffled connection distributions (horizontal lines with carets) and experimentally observed connections (circles) for functional subtypes in M1 (above) and S1 (below). **g**, (Left) Example cell pair receiving common input for each region (top left - visual, top right - auditory, bottom left - motor, bottom right - somatosensory). (Right) Shuffled common input distributions (horizontal lines with carets) and experimentally observed excitatory common input-receiving pairs (circles) for functional subtypes in visual (top left), auditory (top right), motor (bottom left) and somatosensory (bottom right) cortices. Significance thresholds were calculated and set as in panel **a.** Source data are provided as a Source Data file.

Supplementary Table 1

Pie chart values (proportion of all recorded cells) from Figure 2											
Region	% cells	% cells	% cells	% cells	% cells	% cells	% cells	% cells	% cells	% cells	% cells
Visual	12.1	2.1	9.1	12	2.1	1.4	0.4	0.1	21.7	0.8	38.1
Auditory	12.5	2.9	21.3	6.1	2.7	2.7	0	0	15.2	0	36.7
Motor	11.9	3	4.4	9.3	9.7	15	0.5	1.4	23.6	0	21.2
Somato.	6.1	0.5	0.4	0.3	11.6	14.5	0.9	0.1	17	0	48.6

Polar chart values (proportion of cells tuned to at least one covariate) from Figure 2																							
Region	%	%	%	%	%	%	%	%	%	%	%	%	%	%	%	%	%	%	%	%	%	%	%
Visual	2.44	7.04	5.96	1.96	2.6	2.96	5.84	2.04	7.16	2.36	17.07	3.72	0.36	1	0.96	2.8	3.72	2.84	14.07	4.6	7.76	0.4	0.36
Auditory	3.14	7.48	6.39	1.19	2.82	2.93	8.99	0.43	13.11	2.82	11.27	4.01	0.43	1.19	3.03	1.63	5.74	3.47	9.32	2.28	8.13	0.22	0
Motor	2.47	4.86	6.07	1.91	2.81	3.01	3.09	0	3.06	3.79	6.04	3.65	1.07	3.51	8.49	4.13	14	6.46	11.33	3.51	6.69	0	0
Somato.	1.32	6.25	2.64	0.72	1.2	0.12	1.32	0	0.96	0	1.68	0.24	1.44	1.92	14.06	6.85	20.19	7.93	15.14	5.41	10.58	0	0

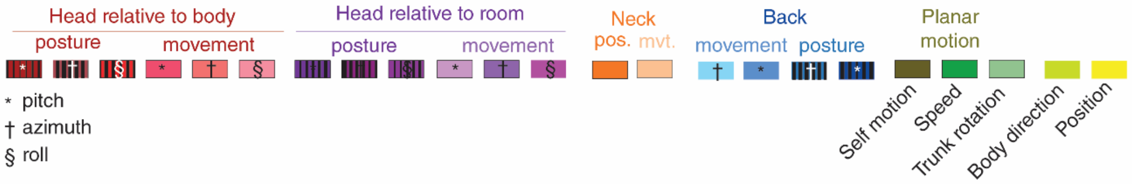

Supplementary Table 1: Numerical distribution of behavioral features encoded in each cortical region. (Top) The fraction of single units in visual, auditory, somatosensory and motor cortices that incorporated specific behavioral features as the first covariate (largest increase of the mean cross-validated relative log-likelihood ratio (rLLR) relative to the null-model) in model selection (refer to the color-coded legend at bottom for feature identification). (Bottom) The percentages of single units statistically linked to one or any larger number of behavioral covariates in each cortical region.

Supplementary Table 2

Statistical comparisons between "weight" and "light" sessions																														
Motor cortex	Head azimuth						Head roll						Head pitch						Speed						Neck elevation					
	AUC		InfoRate		Stability		AUC		InfoRate		Stability		AUC		InfoRate		Stability		AUC		InfoRate		Stability		AUC		InfoRate		Stability	
	z	p	z	p	z	p	z	p	z	p	z	p	z	p	z	p	z	p	z	p	z	p	z	p	z	p	z	p	z	p
Posture	2.54	.05	4.1	2e-5	2.03	.02	-.8	.21	.28	.38	1.0	.15	1.34	.09	3.6	.09	1.63	.05	-3.67	1e-4	3.16	7e-4	2.35	9e-3	-.05	.48	.43	.33	1.81	.03
Movement	.41	.34	3.18	7e-4	3.4	3e-4	.86	.19	1.54	.06	1.51	.06	2.0	.02	.87	.19	2.06	.02	-1.57	.06	-.25	.4	1.04	.14	1.85	.03	1.19	.01	1.16	.12

Statistical comparisons between "weight" and "light" sessions																														
Visual cortex	Head azimuth						Head roll						Head pitch						Speed						Neck elevation					
	AUC		InfoRate		Stability		AUC		InfoRate		Stability		AUC		InfoRate		Stability		AUC		InfoRate		Stability		AUC		InfoRate		Stability	
	z	p	z	p	z	p	z	p	z	p	z	p	z	p	z	p	z	p	z	p	z	p	z	p	z	p	z	p	z	p
Posture	-.3	.38	2.27	.01	.9	1e-3	-1.52	.06	1.82	.03	3.36	3e-4	1.52	.06	1.88	.03	1.3	.09	-3.09	9e-4	1.88	.03	1.36	.09	1.3	.1	.1.08	.14	0.19	.42
Movement	-.61	.27	.86	-.19	1.7	.04	-.86	.19	.42	.33	.89	.19	1.8	.03	-.39	.08	1.04	.19	-1.28	.1	-1.46	.07	1.95	.02	1.44	.07	1.59	.06	.27	.39

Supplementary Table 2: Summary of statistical comparisons between "weight" and "light" sessions. (Top) Details of statistical testing procedures in relation to the area under the curve (AUC), information rate and stability across weight and light sessions in motor cortex as shown in Supplementary Fig. 16d. (Bottom) Same, but for visual neurons. Significance was determined by comparing observed values against a shuffled distribution generated by pseudorandomly permuting session identities of the data 1000 times and recomputing the differences. "z" refers to z-score and "p" refers to p-value in comparison to an empirical shuffle distribution; all comparisons were two-sided.
