# Supplementary material for: Behavioral decomposition reveals rich encoding structure employed across neocortex in rats
Source: Nat Commun. 2023 Jul 4;14:3947. doi: 10.1038/s41467-023-39520-3 (PMC10319800; doi:10.1038/s41467-023-39520-3)
Supplement: Supplementary file 3 — Description of Additional Supplementary Files [file 41467_2023_39520_MOESM3_ESM.pdf]

File name: Supplementary Movie 1

Description: Animation of a digitally rendered rat, portrayed by the head, back (3 blue spheres), and neck (smaller green sphere), demonstrating tuning selectivity of a visual cortical neuron, in darkness, to the action “walk, head left”, and a lack of responsiveness for the non-preferred action, “walk, head right”. The session is fast-forwarded between bouts of the specified action and slowed down when the animal is within-behavior; within-behavior bouts are indicated by a green square, both in this and subsequent videos. The translational components of movement have been removed for visualization purposes, though the animals are freely moving.

File name: Supplementary Movie 2

Description: Same as previous movie, but for a neuron in auditory cortex firing selectively during the action “walk, clockwise head roll”; the non-preferred action is “running, head level”.

File name: Supplementary Movie 3

Description: Example of a motor cortical neuron tuned to the action “hunched, head left”, but not responsive to “hunched, head right”.

File name: Supplementary Movie 4

Description: A cell from primary somatosensory cortex firing selectively during bouts of “rearing”, but not “running, head up”.

File name: Supplementary Movie 5

Description: Example of a visual cortical neuron recorded in darkness, tuned to the action “back hunch”, but not “head right”.

File name: Supplementary Movie 6

Description: A neuron in auditory cortex firing selectively during “rearing”, but not during the action “running head up”.
