# Supplementary material for: Behavioral decomposition reveals rich encoding structure employed across neocortex in rats
Source: Nat Commun. 2023 Jul 4;14:3947. doi: 10.1038/s41467-023-39520-3 (PMC10319800; doi:10.1038/s41467-023-39520-3)
Supplement: Supplementary file 10 — Reporting Summary [file 41467_2023_39520_MOESM10_ESM.pdf]

nature portfolio

Corresponding author(s): Bartul Mimica, Jonathan R. Whitlock

Last updated by author(s): May 23, 2023

Reporting Summary

Nature Portfolio wishes to improve the reproducibility of the work that we publish. This form provides structure for consistency and transparency in reporting. For further information on Nature Portfolio policies, see our Editorial Policies and the Editorial Policy Checklist.

Statistics

For all statistical analyses, confirm that the following items are present in the figure legend, table legend, main text, or Methods section.

n/a	Confirmed	
☐	☒	The exact sample size (*n*) for each experimental group/condition, given as a discrete number and unit of measurement
☐	☒	A statement on whether measurements were taken from distinct samples or whether the same sample was measured repeatedly
☐	☒	The statistical test(s) used AND whether they are one- or two-sided<br>*Only common tests should be described solely by name; describe more complex techniques in the Methods section.*
☐	☒	A description of all covariates tested
☐	☒	A description of any assumptions or corrections, such as tests of normality and adjustment for multiple comparisons
☐	☒	A full description of the statistical parameters including central tendency (e.g. means) or other basic estimates (e.g. regression coefficient) AND variation (e.g. standard deviation) or associated estimates of uncertainty (e.g. confidence intervals)
☐	☒	For null hypothesis testing, the test statistic (e.g. *F*, *t*, *r*) with confidence intervals, effect sizes, degrees of freedom and *P* value noted<br>*Give P values as exact values whenever suitable.*
☒	☐	For Bayesian analysis, information on the choice of priors and Markov chain Monte Carlo settings
☒	☐	For hierarchical and complex designs, identification of the appropriate level for tests and full reporting of outcomes
☐	☒	Estimates of effect sizes (e.g. Cohen's *d*, Pearson's *r*), indicating how they were calculated

Our web collection on statistics for biologists contains articles on many of the points above.

Software and code

Policy information about availability of computer code

Data collection

Commercial software: Motive (OptiTrack) version 2.2.0
Open-source software: SpikeGLX (https://billkarsh.github.io/SpikeGLX) versions 20190724 and 20190919

Data analysis

Commercial software: MATLAB (MathWorks) version r2020b, Python version 3.7

Open-source code (MATLAB):
Kilosort version 2.5 (https://github.com/MouseLand/Kilosort)
UMAP version 1.4.1 (https://www.mathworks.com/matlabcentral/fileexchange/71902-uniform-manifold-approximation-and-projection-umap)

Open-source code (Python, Python packages):

numba	0.53.1
numpy	1.21.5
opencv-python	4.1.1.26
pandas	1.3.5
pycwt	0.3.0a22
PyWavelets	1.3.0
scikit-image	0.19.2
scikit-learn	0.24.2
scipy	1.7.3
sklearn	0.0

statsmodels	0.13.2
umap-learn	0.4.2
networkx	2.6.3
matplotlib	3.5.1
matplotlib-inline	0.1.2
seaborn	0.11.2

Custom generated code used for data acquisition and preprocessing is publicly available and can be found at: https://github.com/bartulem/KISN-PyLab. Code used to analyze the data and make the figures is also public and can be found at: https://github.com/bartulem/KISN-pancortical-kinematics.

Both repositories are linked in the "Code Availability" section of the manuscript.

For manuscripts utilizing custom algorithms or software that are central to the research but not yet described in published literature, software must be made available to editors and reviewers. We strongly encourage code deposition in a community repository (e.g. GitHub). See the Nature Portfolio guidelines for submitting code & software for further information.

Data

Policy information about availability of data

All manuscripts must include a data availability statement. This statement should provide the following information, where applicable:

- Accession codes, unique identifiers, or web links for publicly available datasets
- A description of any restrictions on data availability
- For clinical datasets or third party data, please ensure that the statement adheres to our policy

All datasets generated for this project, together with supporting documentation, are linked in the manuscript and can be downloaded at: https://figshare.com/articles/dataset/Rat_3D_Tracking_E-Phys_KISN_2020_Dataset/17903834

Human research participants

Policy information about studies involving human research participants and Sex and Gender in Research.

Reporting on sex and gender	N/A
Population characteristics	N/A
Recruitment	N/A
Ethics oversight	N/A

Note that full information on the approval of the study protocol must also be provided in the manuscript.

Field-specific reporting

Please select the one below that is the best fit for your research. If you are not sure, read the appropriate sections before making your selection.

☒ Life sciences ☐ Behavioural & social sciences ☐ Ecological, evolutionary & environmental sciences

For a reference copy of the document with all sections, see nature.com/documents/nr-reporting-summary-flat.pdf

Life sciences study design

All studies must disclose on these points even when the disclosure is negative.

Sample size	No sample size calculation was performed. The number of animals was set a priori to at least three per recorded brain area, and the cell yield from each area included all available cells that matched the classification criteria for being well-isolated (independent) from other recorded cells. The data sets obtained were sufficiently large to perform unbiased statistical analyses.
Data exclusions	Only single-unit data were considered in the study. "Noise" clusters (i.e. signals arising from a non-neural origin, such as chewing or contact of the head implant against the arena wall) were separated from single-units and multiunit activity. Multi-unit activity (as defined by inter-spike interval distributions, waveform features and the value of the Kilosort contamination parameter) were excluded from analysis. It is common practice among in vivo electrophysiological studies to exclude noise and multi-unit activity, as this impedes the study of tuning properties exhibited by well-isolated neurons.
Replication	The forms of neural tuning reported in the study (for posture and movement of the head and body) were observed in all recording sessions from all animals in each group. Groups consisted of animals with probes implanted in visual and auditory cortices (n = 3 rats) or in the somatosensory and motor cortices (n = 4). Data sets from each rats consisted of 20 minute recordings collected during 7-8 separate sessions.

Randomization	The study contained no randomization to experimental treatments. This is because all animals were of the same species, strain, body weight, and had similar behavioral habituation. The same recording room and recording systems were used for all animals.
Blinding	Blinding for data analysis was not necessary because neural recordings from all experimental conditions were processed and analyzed concurrently and in the same batches, and because the same animals were used in the different experimental conditions.

Reporting for specific materials, systems and methods

We require information from authors about some types of materials, experimental systems and methods used in many studies. Here, indicate whether each material, system or method listed is relevant to your study. If you are not sure if a list item applies to your research, read the appropriate section before selecting a response.

Materials & experimental systems

n/a	Involved in the study
☐	☒ Antibodies
☒	☐ Eukaryotic cell lines
☒	☐ Palaeontology and archaeology
☐	☒ Animals and other organisms
☒	☐ Clinical data
☒	☐ Dual use research of concern

Methods

n/a	Involved in the study
☒	☐ ChIP-seq
☒	☐ Flow cytometry
☒	☐ MRI-based neuroimaging

Antibodies

Antibodies used	Guinea pig-derived anti-NeuN primary antibody (1:1000 dilution) catalog no. ABN90P, Sigma-Aldrich, USA.
Validation	Sigma-Aldrich provides the following quality assessments:<br><br>Evaluated by Western Blot in mouse E16 brain tissue lysate.<br>Western Blot Analysis: A 1:1,000 dilution of this antibody detected NeuN on 10 µg of mouse E16 brain tissue lysate.

Animals and other research organisms

Policy information about studies involving animals; ARRIVE guidelines recommended for reporting animal research, and Sex and Gender in Research

Laboratory animals	A total of 7 male Long-Evans rats (age: 3-4 months, weight: 350-450 g) were used in this study.
Wild animals	No wild animals were used in the study.
Reporting on sex	The sex of the animals was not considered in this study because the forms of neural tuning investigated (for body posture, movement and self-motion) occur the same in male and female animals.
Field-collected samples	No field collected samples were used in the study.
Ethics oversight	Experiments were performed in accordance with the Norwegian Animal Welfare Act and the European Convention for the Protection of Vertebrate Animals used for Experimental and Other Scientific Purposes. Protocols were approved by the Norwegian Food Safety Authority (FOTS ID 13511).

Note that full information on the approval of the study protocol must also be provided in the manuscript.
